# How typhoons trigger turbidity currents in submarine canyons

**DOI:** 10.1038/s41598-019-45615-z

**Published:** 2019-06-25

**Authors:** Octavio E. Sequeiros, Michele Bolla Pittaluga, Alessandro Frascati, Carlos Pirmez, Douglas G. Masson, Philip Weaver, Alexander R. Crosby, Gianluca Lazzaro, Gianluca Botter, Jeffrey G. Rimmer

**Affiliations:** 1 Shell Global Solutions International B.V., Lange Kleiweg 40, 2288 GK Rijswijk, The Netherlands; 2 Now at University of Genova, Department of Civil, Chemical and Environmental Engineering, Via Montallegro 1, 16145 Genova, Italy; 3 Shell Global Solutions International B.V., Grasweg 31, 1031 HW Amsterdam, The Netherlands; 4 Shell Exploration & Production Company, 150 N. Dairy Ashford, 77079 Houston, USA; 5 Seascape Consultants, Jermyns House, Jermyns Lane, Romsey, SO51 0PE UK; 6 Oceanweather Inc., 350 Bedford Street, Suite 404, Stamford, CT 06901 USA; 7 University of Padova, Department of Civil Architectural and Environmental Engineering. Via Loredan 20, 35131 Padova, Italy; 8 i4 consulting s.r.l. Via Barroccio del Borgo 1, 35124 Padova, Italy; 9 Shell Philippines Exploration BV, P.O. Box 171, Ayala Alabang, Muntinlupa City, 1780 Manila, Philippines

**Keywords:** Natural hazards, Atmospheric dynamics, Physical oceanography, Hydrology, Power distribution

## Abstract

Intense turbidity currents occur in the Malaylay Submarine Canyon off the northern coast of Mindoro Island in the Philippines. They start in very shallow waters at the shelf break and reach deeper waters where a gas pipeline is located. The pipeline was displaced by a turbidity current in 2006 and its rock berm damaged by another 10 years later. Here we propose that they are triggered near the mouth of the Malaylay and Baco rivers by direct sediment resuspension in the shallow shelf and transport to the canyon heads by typhoon-induced waves and currents. We show these rivers are unlikely to generate hyperpycnal flows and trigger turbidity currents by themselves. Characteristic signatures of turbidity currents, in the form of bed shear stress obtained by numerical simulations, match observed erosion/deposition and rock berm damage patterns recorded by repeat bathymetric surveys before and after typhoon Nock-ten in December 2016. Our analysis predicts a larger turbidity current triggered by typhoon Durian in 2006; and reveals the reason for the lack of any significant turbidity current associated with typhoon Melor in December 2015. Key factors to assess turbidity current initiation are typhoon proximity, strength, and synchronicity of typhoon induced waves and currents. Using data from a 66-year hindcast we estimate a ~8-year return period of typhoons with capacity to trigger large turbidity currents.

## Introduction

## Typhoons as Triggers of Turbidity Currents

Tropical cyclones such as hurricanes and typhoons regularly devastate islands and coastal regions taking enormous toll in fatalities and property^1–3^, affecting large scale precipitation^4–6^ and ocean circulation^7,8^ patterns. Less known is that they can directly trigger submarine mass gravity flows due to the waves, currents and surges they induce. Cyclones can also trigger flows indirectly, with a small lag after their passage, because of peak flood discharges in rivers. They can also trigger flows after a longer delay, days to months or even years, as a result of slope instability and failure of large volumes of rapidly deposited sediment near the river mouth^9^.

Typhoons frequently pass over the Philippines and the entire northwestern Pacific. Indirect triggering of turbidity currents has been observed in the Gaoping Canyon offshore Taiwan, borne out of hyperpycnal flows generated by peak flood discharges associated with typhoon rainfall^10,11^. In the Philippines there are cases of indirect mass gravity flow triggers in the Cagayan Canyon associated to typhoon Kadiang in 1993, and typhoon Nanmadol in 2004^9^.

Evidence of direct triggering is more elusive. During major storms, pulses of downcanyon near-bottom sediment transport have been observed at the Cap de Creus Submarine Canyon^12^.

There are examples of cable breaks caused by gravity flows directly generated by tropical cyclones in Chilung Canyon, Taiwan; Mafate and Saint-Denis Fans, La Reunion; and Izu-Bonin Ridge on the continental slope offshore Japan^9^. Submarine gravity flows also appear to have been triggered during the passage of hurricanes near the Mississippi River mouth, but whether such flows were triggered by landslides, wave/current conditions or by hyperpycnal flows is unclear.

Direct resuspension of shelf sediment by waves and currents deposited around and within canyon heads, combined with sediment transport by currents into the canyon, is a plausible mechanism for creating the suspended sediment concentration necessary to generate a turbidity current. Wave-induced thin near-bed sandy gravity flows are known to occur in shallow waters^13^.

Direct resuspension of sediment can occur in tandem with slope failure and run-out due to dynamic loading of the seafloor^9^. Dynamic loading is the result of storm waves, currents and surges during tropical cyclones exerting large hydrodynamic pressures on the seafloor and elevating subsurface pore pressures^14–18^. Wave crests increase pore pressures, while wave troughs generate seepage pressures^19^. Eventually this can cause liquefaction or the rupture of inter-particle cohesive bonds leading to sediment failure^20^. In addition, the orbital motion of water particles exerts horizontal shear on the seabed^21^. Failure and sediment transport can occur by shear, liquefied flow or slope failure^22^. Horizontal shear stresses induced by cyclone-forced currents can induce failure of weak sediments in the same way^23^. Slope failure attributed to dynamic loading also occurs well below the wave action line as deep as 1200 m^9^.

In this paper we investigate the nature of two large turbidity currents that within a 10-year period impacted upon a submarine pipeline offshore Philippines near the mouth of the Malaylay and Baco rivers. These rivers have relatively small but high relief drainage basins and debouch into an area with a very narrow shelf and steep slope. The area is known for significant tectonic activity, monsoon rains and the passage of strong typhoons. The turbidity currents were detected after they occurred, during regular monitoring activities of the pipeline, which evidenced lateral displacement and erosion of the surrounding sea-floor, and damage of a shielding rock berm. Significant typhoons had occurred in the months prior the monitoring surveys, but the exact timing, trigger mechanism and physical processes of the submarine events that resulted in pipeline displacement remained elusive. We first describe the observations from seabed surveys, meteorological and oceanographic conditions. These data informed the quantitative modelling of a) fluvial discharge in the river catchment basins, b) typhoon-induced wave/current conditions in the coastal areas, and c) turbidity currents in the canyons. This source-to-sink integrated approach provides a basis to predict future events, and to estimate their impact on the seafloor near the existing pipeline, thus enabling engineering solutions to protect the environment from the consequences of damage to critical seafloor infrastructure.

## Results

Results rely on a wide array of data and models: repeat high resolution bathymetric surveys, sub-bottom profiles, and sediment core data; rainfall and meteorological data from nearby weather stations, a rainfall-runoff model for the Malaylay and Baco rivers; typhoon tracks and a dedicated typhoon hindcast for Verde Island Passage, and a three-dimensional turbidity current model. Further details of data and analysis are found in Methods and Supplementary sections respectively.

### Turbidity currents in the Malaylay Submarine Canyon

**Figure 1:**
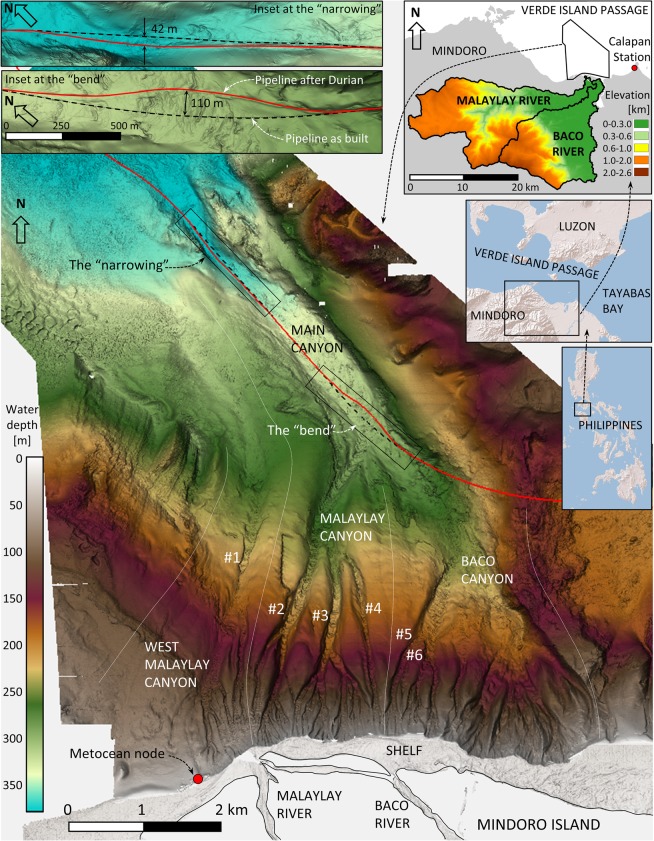
Bathymetry map of the Malaylay and Baco Submarine Canyons, and their merging into the larger Main Canyon in Verde Island Passage. Black dashed line is the pipeline as built. Red line is the pipeline now. Red dot indicates position of waves and current hindcast node at the shelf break. Relevant tributary channels are named #1–6. The “bend” is the area where the MSC merges into the MC. The “narrowing” is an area of reduced width in the MC. Insets indicate submarine canyons location in VIP, the river drainage basins, and details of the pipeline displacements.

The Malaylay and Baco Submarine Canyons (MSC, BSC) are located off the northern coast of Mindoro Island in the Verde Island Passage (VIP), a strait between Luzon and Mindoro Islands. The Malaylay and Baco rivers debouch straight into the canyons heads in an area with a very narrow continental shelf (~300 m wide). Multiple submarine canyons on the upper slope coalesce further downstream into a larger Main Canyon (MC). A pipeline is located on the seabed of the MC (Fig. 1).

Since its installation in 2001 the gas pipeline, whose external diameter is 72 cm, has experienced two large mass gravity flow events in 2006 and 2016. The timing of the events corresponds to the nearby passing of major typhoons and strongly suggests a cause and effect relationship, but details of the triggering mechanisms and initiation of the flows remained elusive.

The first event was detected during a pipe survey in early 2007. Two pipe displacements were found, the first 110 m off its axis in the area known as the “bend” where the MSC merges into the MC; and another of 42 m in the “narrowing” further downstream in the MC. It was then conjectured that the passage of typhoon Durian in November 2006 may have been related to the pipeline displacements. The second event, recorded by a survey in early 2017 after the passage of typhoon Nock-ten in December 2016, severely damaged the rock berm that had been put in place to protect the pipeline after the 2006 event. Damage occurred in the same two locations where the pipeline had been earlier displaced. The exact timing of the mass gravity flows is unknown because the pipeline was not ruptured but can be constrained by the surveying and monitoring activities of the pipeline as indicated in Table 1.

**Table 1:** Sequence of surveys and typhoons relevant to VIP.

Date	Survey*/TYPHOON^†^
Jan–Nov 1997	Pre-installation pipe survey
Apr 2001	As-built survey
May 2004	Pipe survey
May 2005	Pipe survey
Apr 2006	Pipe survey
Sep 2006	XANGSANE
Nov 2006	DURIAN
Dec 2006	UTOR
Apr 2007	Pipe survey when displacement was found
Jun 2007	Area survey, sediment coring.
Mar 2008	Pipe survey
Jun 2008	FENGSHEN
Jun–Jul 2008	Area survey
Apr 2009	Pipe survey
Jun–Jul 2009	Pre/post rock berm
May–Jun 2010	Pipe survey
Jun–Jul 2011	Pipe survey
Nov 2011	Area survey
Mar 2012	Pipe survey
Mar–Apr 2013	Pipe survey
Oct 2013	NARI
Nov 2013	HAIYAN
Mar–May 2014	Pipe survey
Jul 2014	RAMMASUN
Dec 2014	HAGUPIT
Jan–Mar 2015	Pipe survey
Jul–Aug 2015	Area survey
Dec 2015	MELOR
Feb 2016	Pipe survey
Sep–Nov 2016	Area survey, sediment coring
Nov 2016	TOKAGE
Dec 2016	NOCK-TEN
Mar 2017	Pipe survey when rock berm damage was found
Jun 2017	Area survey, sediment coring
Sep 2017	Pipe survey
Feb2018	Pipe survey
Aug 2018	Pre/post rock berm
Sep 2018	Pipe survey

*Pipeline surveys are carried out by Remotely Operated Vehicle (ROV) and limited to the pipeline surroundings; larger bathymetric area surveys are usually done by Autonomous Underwater Vehicle (AUV) and extend up to the MSC. ^†^Only typhoons relevant for VIP are listed.

Evaluations carried out after the first event showed that mass gravity flows, specifically turbidity currents, were the cause of pipeline displacement. The features observed on repeat bathymetric and sonar data, sediment cores and photographs all pointed to turbidity currents as opposed to landslides, debris-flows, or ground motion generated by earthquakes. Yet, there was uncertainty on the ultimate cause, flow duration and recurrence.

In the MSC we can rule out dynamic loading causing slope failure because there is no morphological evidence of any recent slope failure or landslide. On the other hand, there are many factors indicating that metocean and/or hydrological conditions induced by typhoons could trigger turbidity currents. These include: (a) a nearby source of sediment from the Malaylay and Baco rivers, coupled with the major rains associated with the seasonal monsoon and passage of typhoons; (b) shallow waters in the very narrow shelf; (c) abrupt transition from the shelf into the canyon system and; (d) large waves and currents during typhoons.

Accordingly, the likelihood of two processes have been further assessed: (1) indirect triggering because of peak flood discharges in the Malaylay and Baco rivers, a process that is slightly delayed with respect to the typhoon rains, depending on the time required to build up the discharge in the drainage basin and; (2) direct triggering of turbidity currents due to sediment resuspension by typhoon-induced waves, currents and surges. This process is synchronous with the arrival of the waves and currents induced by the typhoon.

### Typhoons as turbidity current trigger in Verde Island Passage

**Figure 2:**
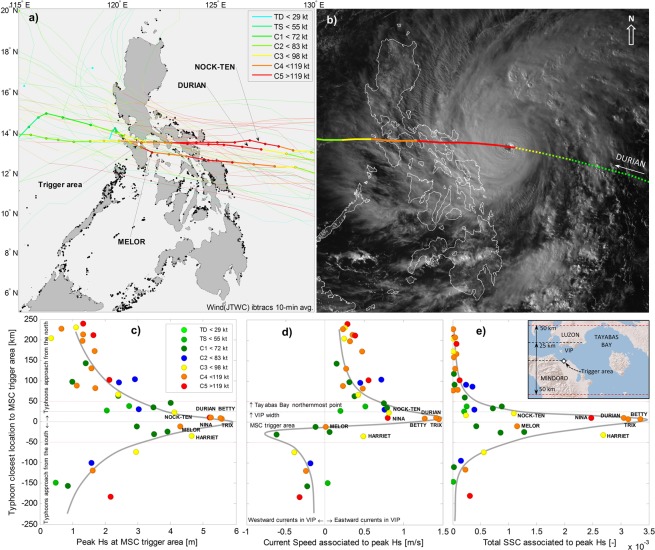
(**a**) Track of relevant tropical cyclones near VIP (IBTrACS, Joint Typhoon Warning Center, JTWC). Colors indicate Saffir-Simpson 10-min sustained-wind scale. Typhoons Durian, Melor and Nock-ten are highlighted with markers at 3-hr intervals. (**b**) Typhoon Durian approaching VIP area on 29/Nov/2006 at 15:30 local time. Source Japan Meteorological Agency via Wikimedia Commons. (**c**) Peak significant wave height, (**d**) associated current speed, and (**e**) total suspended sediment concentration at the typhoon track location closest to the trigger area. Horizontal dashed lines indicate characteristic distances from the MSC trigger area, as indicated in the inset. Gray thick lines are estimated fit of the data.

Between 1951 to the end of 2016 there have been about 40 typhoons that passed within 200 km of VIP, all of which have been included in a hindcast analysis (Fig. 2a, Table S1). Typhoons typically move from the Pacific Ocean predominantly with a west-northwest trajectory. As they enter the Philippines archipelago their strength can diminish due to interaction with the rough topography and changes in the underlying water temperature. However, typhoons can arrive in VIP still at their strongest form as there is only about 300 km to the easternmost islands of the Philippines, a distance travelled by a typhoon’s eye in about one day.

Proximity of the typhoon is arguably the main factor in defining metocean conditions in VIP. Based on hindcast and typhoon track data, the closer the typhoon path to VIP is, the larger the wave height is. Strength of the typhoon, defined by its maximum sustained wind speed, is the second most important factor. Typhoons that pass within 50 km of the trigger area (i.e. the shelf break at the MSC head) and are category 3 or higher, induce significant wave heights above 4 m and resuspend much sediment (Fig. 2c–e). Regardless of strength, capacity to resuspend sediment drops fast when typhoons pass more than 50 km away from the MSC trigger area.

**Figure 3:**
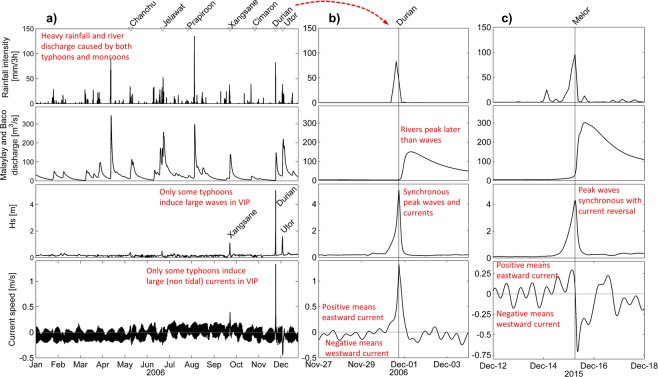
(**a**) Rainfall intensity at Calapan, Malaylay and Baco rivers discharge, significant wave height and current speed at MSC trigger area during 2006. Detailed time series of typhoons (**b**) Durian and (**c**) Melor. Typhoons names are indicated above panels. See Fig. 1 for locations of Calapan station and the wave and current node.

Current speeds induced by typhoons are also stronger when the path of the storm is closer to VIP and can exceed tidal currents by an order of magnitude. When typhoons pass to the north of VIP currents flow toward the east in the passage. When typhoons pass to the south of VIP currents flow toward the west. This occurs primarily due to the counter-clockwise wind pattern of tropical cyclones in the northern hemisphere, and the narrow VIP orientation along an east-west axis (Fig. 1). Typhoons passing very close to VIP can create currents which reverse direction (i.e. currents speed is zero) almost synchronous with the peak waves (Fig. 3).

We estimate that during some typhoons the combined action of waves and currents in the shallow waters of the shelf (<15 m) has enough capacity to resuspend and move sand during substantial parts of the storm (Methods: Typhoon-induced waves and currents). Once suspended sediment gets to the steep head of the MSC, the excess density of the suspension initiates the flow of a turbidity current. Subsequent entrainment of sediment previously deposited on the canyon floor likely results in flow acceleration and ignition downstream^24–27^.

### Likelihood of hyperpycnal flows from Malaylay and Baco rivers

Submarine turbidity currents can be initiated by transformation of turbid river plumes into hyperpycnal flows^28^. Hyperpycnal flows can be of long-duration (i.e., days to weeks) concomitant with the duration of the river flood. Knowledge of the rating curve relating sediment and water discharge at the river mouth is a key requirement to predict frequency and duration of hyperpycnal flows. Such information is not available for the Malaylay and Baco rivers, which have no monitoring stations.

The rainfall-runoff model (Methods: Hyperpycnal assessment) indicates that heavy floods are induced by rainfall events when the antecedent moisture of the catchment is relatively high. While highest river discharges during 2000–2017 are explained by both monsoon and typhoon induced rains, the former are more dominant (Fig. 3). Return periods of the floods associated with typhoons Durian (2006) and Nock-ten (2016) turn out to be rather limited (5 years) in terms of peak flows.

The BQART predictor^29^ estimates yearly averaged sediment delivery of the Baco-Malaylay rivers to the ocean as a function of mean annual water discharge and available basin-wide lithological, physiographic, and climatic data. BQART predictions indicate that the Baco and Malaylay rivers have a sediment yield of 0.336 and 0.351 MT/year respectively.

Coupled sediment transport and hydrological modelling show that maximum suspended sediment concentrations at the river mouths during 2000–2017 floods were never high enough to generate hyperpycnal flows (Supplementary Information: Hydrology). In addition, there is a mismatch between the high frequency of monsoon floods (several events per year), and the sporadic nature of large turbidity currents. Monsoons do not induce large waves or currents in VIP. We can also rule out a combined trigger during typhoons because the Malaylay and Baco rivers peak at the mouth about 9–12 hours later than waves. By the time the peak discharge comes into VIP waves are mostly gone (Fig. 3b,c). Therefore, the turbidity currents in 2006 and 2016 were most probably not triggered by hyperpycnal flows.

### Critical typhoons: the significance of Durian, Melor and Nock-ten

Since the installation of the pipeline in 2001 there have been about eleven typhoons passing over VIP area or nearby, and two large turbidity currents based on repeat surveys of the pipeline. Hence, not all typhoons trigger noteworthy turbidity currents. Three large typhoons that passed in close vicinity to VIP deserve especially attention: Durian (Nov 2006), Melor (Dec 2015) and Nock-ten (Dec 2016). We find that Durian and Nock-ten passed over the area just before surveys detected a displaced pipeline and damaged rock berm. No noteworthy seabed changes were detected during surveys in 2016 after the passage of Typhoon Melor (Dec 2015). This despite generating wave conditions in VIP larger than Nock-ten (Fig. 2c).

The explanation for this paradox is to be found in the typhoon-induced currents and their reversal under certain typhoon tracks. Typhoons passing north of the MSC trigger area (e.g. Durian, Nock-ten) generate currents and waves that work together to produce conditions under which turbidity currents are initiated; but typhoons to the south of the trigger area (e.g. Melor) do not because of the current reversal at the same time as the peak waves (Fig. 3c). We conjecture that without strong currents acting synchronously with peak waves there is not enough capacity to transport wave-resuspended sediment to the MSC head.

### Recreating Durian and Nock-ten turbidity current events

In agreement with observations, numerical simulations of turbidity currents predict the triggering of large turbidity currents associated with typhoons Durian (2006) and Nock-ten (2016), and the absence of any significant turbidity current associated with Melor (2015). The turbidity current model is fed along the entire length of the MSC head directly with the suspended sediment flux derived from typhoon-induced waves and currents at the shelf break. The sediment flux is defined by the currents speed and direction and the total suspended sediment concentration at the shelf break (Methods: Turbidity current modelling).

**Figure 4:**
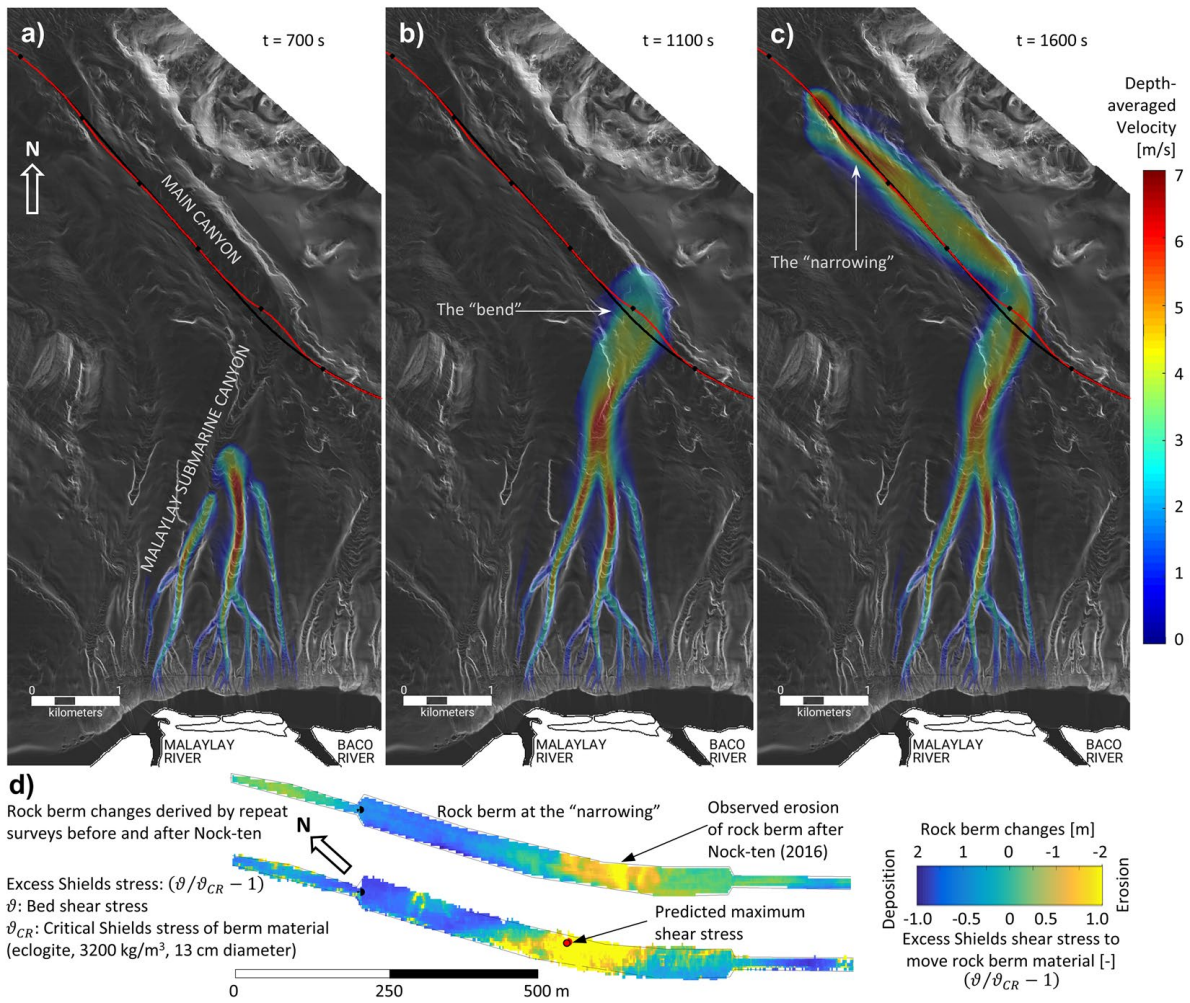
At the top three instances of numerically simulated turbidity current associated with typhoon Durian (Nov. 2006) in MSC. Colors are depth-averaged velocity in m/s. (**a**) In upstream tributary channels. (**b**) Crossing pipeline at the “bend”. (**c**) Further downstream in the “narrowing”. Black line is ‘as built’ pipe, red line is pipeline after Durian. Pipeline deformations at the bend and the narrowing are coincident with the crossing of the turbidity current. (**d**) Observed erosion/deposition map of the rock berm at the “narrowing” based on repeat surveys, and modelled excess Shields stress by the turbidity current associated with typhoon Nock-ten (Dec. 2016). Stress is relative to the critical threshold of transport of rock berm material (eclogite, 3200 kg/m^3^, 13 cm mean diameter). Yellow represents erosion, blue deposition. Red dot marks location where the maximum shear stress is predicted. Berm is stretched x2 in the vertical to improve visualization.

Three stages of the modelled turbidity current triggered by typhoon Durian are shown in Fig. 4. The turbidity current starts as a uniform sheet flow all along the canyon head, but it is rapidly channelized into the multiple small canyons of the upstream MSC system. Through these smaller channels the currents coalesce into larger and larger canyons before merging into the main trunk of the MSC. The high angle of attack of the current with respect the pipeline is believed to be the main reason for the large lateral deformation at the “bend”. The deformation at the “narrowing” is related to flow acceleration as the MC width decreases from 400 m to 150 m in one kilometer, and to a steeper seabed gradient that increases from an average slope of ~1° in the middle of the MC to a value of ~2.8° around the “narrowing”.

Modelled flow velocity and shear stress fields are also consistent with observations that the rock berm was washed away at the “narrowing” during the event associated with Nock-ten (2016). Bed shear stress caused by the turbidity current triggered by typhoon Nock-ten matches very well the observed erosion/deposition pattern and rock berm damage derived from repeat surveys (Fig. 4d).

The agreement between model predictions and observed erosion/deposition patterns buttresses the premise of a turbulence supported flow being the ultimate cause of activity in the submarine canyons.

### Turbidity current signature on the canyon seabed

From sediment seabed cores taken in 2007 and 2017 we observe that the MSC is dominated by coarse-grained sediment (sand and gravel) with a minor component of mud (defined as finer than 64 μm). Sediment is coarser within the channels (d_50_~200–500 μm) and finer on the overbank and inter-channel areas (d_50_~15–80 μm). A similar, albeit finer, sediment pattern is found in the MC. This provides an indication of the characteristic sediment size that is transported by turbidity currents in the MSC/MC system (Supplementary Information: Core data).

Seafloor bedforms are a characteristic feature of the MSC and BSC systems (numbered 1–6, Fig. 1). Bedforms are interpreted to be formed primarily by turbidity currents and are indicative of sediment transport by bedload and/or suspended load. Bedforms are absent along inter-canyon areas on the slope, indicating that turbidity currents become channelized almost as soon as they enter the slope.

**Figure 5:**
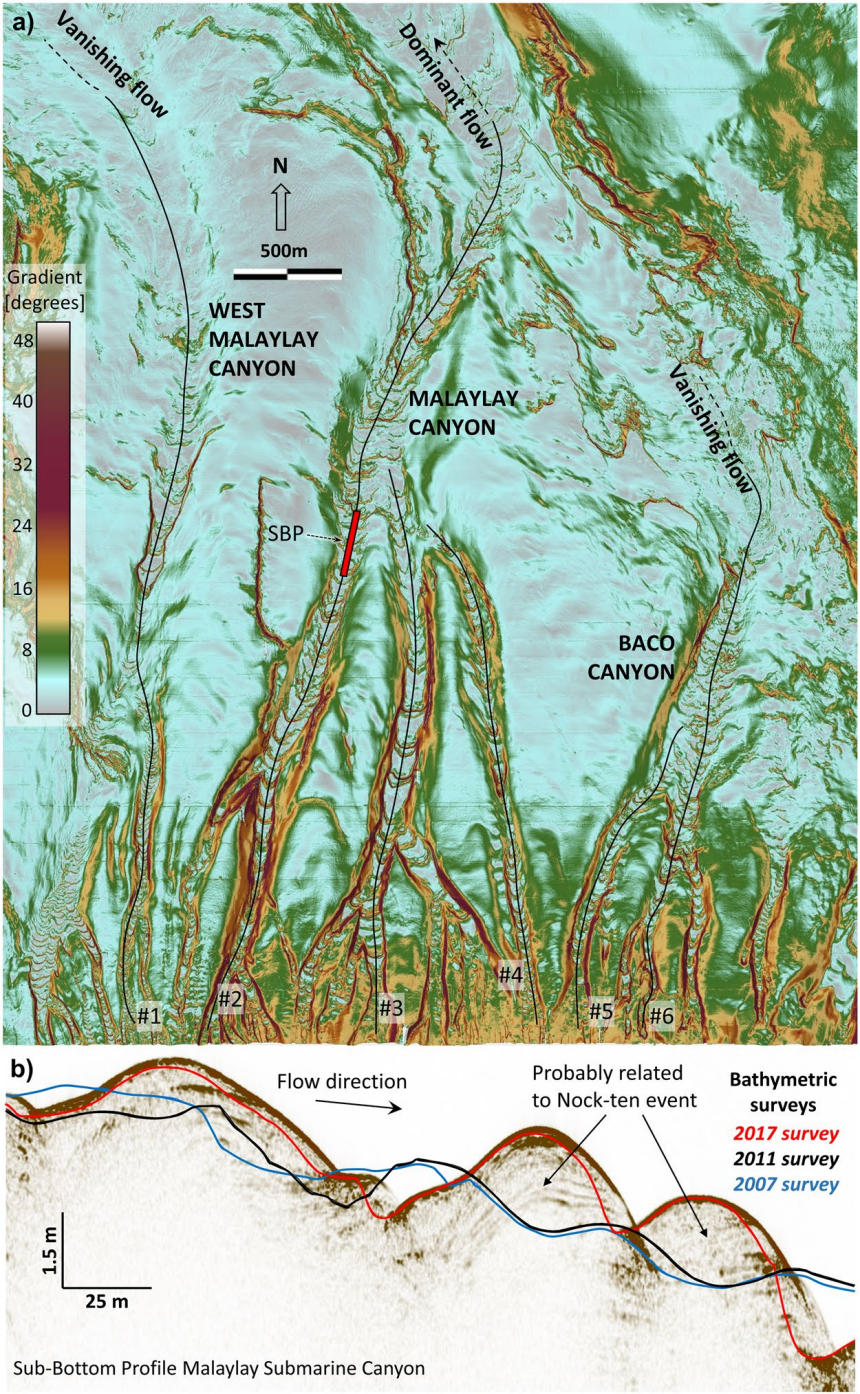
(**a**) 2017 seabed gradient map (degrees) of the main canyons cutting the slope north of the Malaylay and Baco river mouths. Bedforms east of channel #4 die out downslope. (**b**) Segment of Sub-Bottom Profiler (SBP) data along channel #2 revealing internal stratigraphy of bedforms with inclined strata dipping against the flow direction, representing deposition on the stoss side and erosion/by-pass on the lee side. Superimposed bathymetric surveys (coloured profiles) highlight bedform migration between 2011 and 2017 surveys. Nock-ten is believed to be responsible for most of the changes.

Along canyons #2–4 (MSC) bedforms can be observed across the entire slope and continuing beyond, into the MC (Fig. 5a). In the West-MSC and BSC bedforms are present only on the upper reaches, and appear to die out further downslope, suggesting that flows gradually diminish before reaching the base of the slope. Only flows channelized through MSC system are thought to reach the MC undiminished. This is derived from the bedform patterns at the location where the MSC joins the MC.

In the MSC and BSC the shape of the bedforms has a characteristic planimetric curvature with a concave crest facing downstream. This shape suggests the bedforms are migrating upstream (upslope), and that the rate of migration is faster in the thalweg than in the margins. MSC bedforms have wavelengths between 20 and 120 m (average 55 m) and heights of the order of 2–4 m. Repeat bathymetry surveys and sub-bottom profiles provide evidence of intense upstream migration likely during typhoon induced turbidity currents (Fig. 5b).

In the MC region bedforms are present all the way across the seafloor and also migrate upslope. They have a barcan-like form, with semicircular downslope elongated shape and downstream concavity. Bedform wavelength range is 100–200 m and height 1.5–2 m.

Based on their dimensions, migration patterns and estimated bankfull flow dimensions we believe these bedforms are most likely anti-dunes^30^, however they may also be cyclic steps associated with transcritical flow behaviour^31^.

## Discussion

### Ignition boundary of large turbidity currents

Turbidity current modelling of the entire waxing and waning phases of the most relevant typhoons enabled identification of the ignition boundary. This is the threshold of inlet conditions at the canyon head above which significant turbidity currents are triggered in the MSC. The criteria to define ignition was the occurrence of a significant turbidity current in at least one of the main three upstream branches of the MSC system. By significant it is meant flow velocities above 1 m/s (to stand above tidal and oceanic currents observed in VIP) and that the triggering happens in a relative short time, less than 30 min.

**Figure 6:**
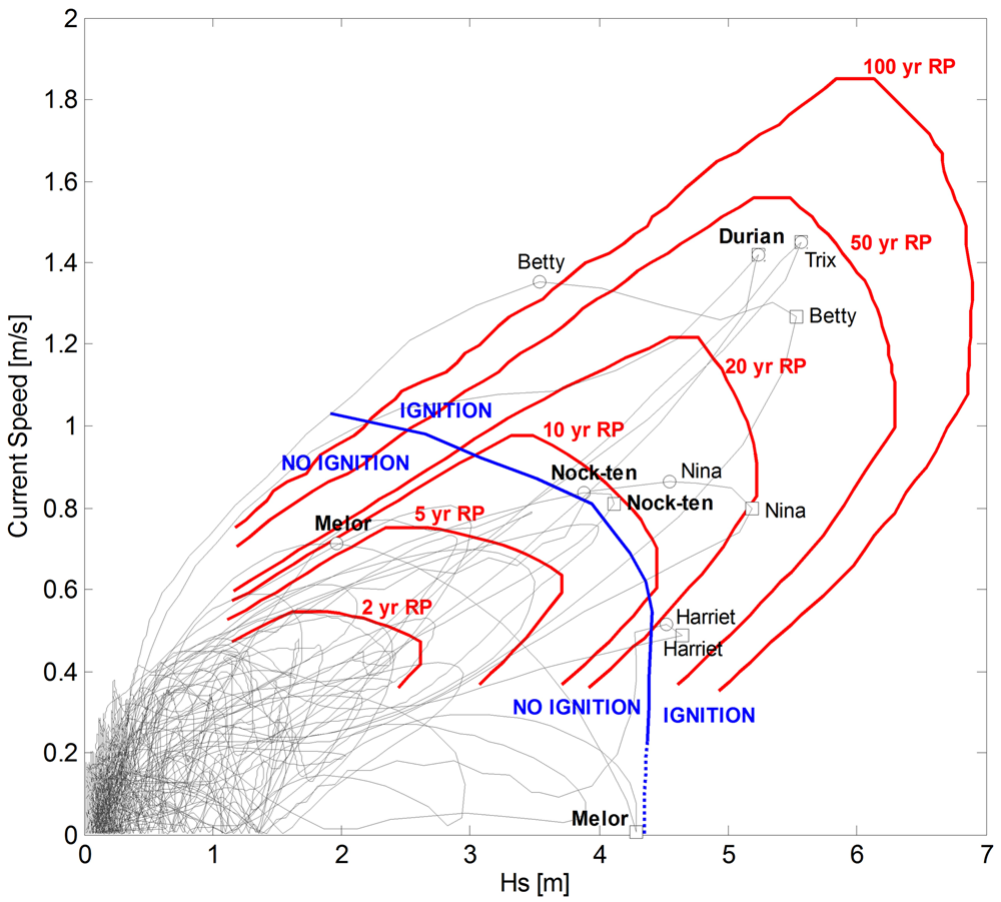
Ignition boundary and joint extreme value analysis based on significant wave height and current speed at the shelf break. Gray lines represent the tracks of the entire typhoon dataset. White squares and circles indicate peak significant wave height (Hs) and associated current speed (CS), and peak CS associated Hs respectively for some selected typhoons. Blueline is the ignition boundary for triggering large turbidity currents in the Malaylay Submarine Canyon. Red lines are joint return period contours. Six typhoons are estimated to have triggered large turbidity current events in the 1951–2016 period: Trix (1952), Harriet (1959), Betty (1987), Nina (1987), Durian (2006) and Nock-ten (2016).

Figure 6 shows the entire typhoon dataset in a plane defined by the significant wave height and current speed at VIP trigger area. The ignition boundary is shown in blue. In the 66 years of the 1951–2016 period the following six typhoons have most probably ignited significant turbidity current events at the MSC: Trix (1952), Harriet (1959), Betty (1987), Nina (1987), Durian (2006), Nock-ten (2016). Other typhoons may have triggered smaller turbidity currents. The ignition boundary allows a first order estimation of turbidity currents duration (Supplementary Information: Turbidity current modeling).

### Turbidity current recurrence

Both waves and currents play a role in resuspending and moving sediment across the shelf. Hence the triggering and recurrence of turbidity currents is calculated by means of Joint Extreme Value Analysis (JEVA) as proposed by Heffernan and Tawn^32^.

JEVA-derived isolines for different return periods are shown in Fig. 6 in red. At peak conditions Nock-ten has a return period of about 8 years, while Durian is slightly over 30 years.

Melor’s peak significant wave height has a negligible associated current speed. This condition falls outside the area where JEVA is robustly computed. The middle section of the ignition boundary lies below the 10-year return period contour.

There is no significant change in global tropical cyclone numbers since 1970^33,34^, and apart from interdecadal variability related to different phases of the El Niño/Southern Oscillation and the Pacific Decadal Oscillation, no significant change in tropical cyclone numbers in the Philippines^35^. Extreme values and recurrence might have to be updated in the future if relative intensification^33^, poleward migration^36^, translation speed slowdown^37^, or lower activity^38^ eventually change regional typhoon maximum intensity or frequency beyond observed natural variability.

### Is there enough sediment to trigger large turbidity currents?

Sediment supply from the Baco and Malaylay rivers is not a limiting factor for turbidity current triggering. On average the rivers bring annually enough sediment to feed a large turbidity current event all along the canyon head. The frequency of large turbidity currents is more sporadic than once per year. (Supplementary Information: Sediment and sea bed).

### Is the Baco Submarine Canyon also active?

All evidence points to the MSC being more active than the BSC. This is inferred by (a) the location of the pipeline displacement at the “bend” where the MSC merges into the MC, (b) different bedform patterns in MSC and BSC, with bedform migration in the entire MSC and bedforms that die out downslope in the BSC, (c) MSC bedforms dominate the area where canyons meet, with clear cross-cutting relationships indicating more recent activity in the MSC than in the BSC and the upstream part of the MC, and (d) the fact that the tributary channels of MSC system are located in between the Malaylay and Baco rivers, and are therefore exposed more directly to sediment sources than BSC, located further east.

## Conclusions

We studied the root cause and the dynamics of powerful turbidity currents in the Malaylay Submarine Canyon. It is concluded that sediment resuspension and transport into the canyon heads by wave and current action is most likely the trigger for the turbidity currents associated with typhoon Durian in 2006 and typhoon Nock-ten in 2016. This is compatible with evidence of timing of turbidity currents in relation to the typhoons, and of simultaneous flows in multiple channels on the upper Malaylay Submarine Canyon. Geological evidence for recent landslide activity in the mapped area, such as landslide scars, blocky landslide deposits or debris flows, is completely lacking. Instead, an organized set of tributary canyon systems, covered with regular sedimentary bedforms, clearly indicates the passage of turbulent flows carrying sediments in suspension and bedload. It is unlikely that some combination of river flood and wave action contributed to the trigger, since they appear to operate at different times, with peak flood at the river mouth lagging the arrival of large waves and high currents by a few hours.

The three main factors for a typhoon to ignite turbidity currents in the Malaylay Submarine Canyon are proximity, strength, and synchronicity of typhoon induced waves and currents. The synchronicity depends on the typhoon approach latitude with respect to the canyon head. Typhoons approaching the Malaylay Submarine Canyon trigger area from the north such as Durian, induce currents that peak together with the waves. Conversely typhoons approaching it from the south such as Melor, induce currents that reverse direction (i.e. current speed is zero) at a time when waves are at their peak. This reduces the chances of turbidity current ignition.

Turbidity currents in the Malaylay Submarine Canyon system are likely initiated at the shelf break simultaneously in the many smaller uppermost canyons that gradually coalesce downstream into larger canyons before merging into the Malaylay Submarine Canyon proper and later into the Main Canyon.

Turbidity current modelling matches field evidence in the form of pipeline displacement and rock berm damage associated with Durian (2006) and Nock-ten (2016) events and predicts the absence of any significant turbidity current linked to Melor (2015), providing assurance that the physical processes of turbidity current initiation and development are qualitatively and quantitatively well understood.

The average recurrence of a typhoon capable of triggering a large turbidity current is about 11 years. A finer calculation by means of Joint Extreme Value Analysis assigns Nock-ten a return period of 8 years; and Durian’s slightly over 30 years. Smaller turbidity currents probably occur more frequently. The duration of Nock-ten’s turbidity current is estimated to be about 45 minutes, whereas Durian’s was a longer event likely exceeding 3 hours.

Hyperpycnal flows are improbable. The Malaylay and Baco rivers are not expected to have sediment concentrations high enough to result in plunging flows. Moreover, if hyperpycnal flows were the main mechanism, turbidity current should occur at a higher frequency than observed, even on yearly basis associated with monsoon floods.

## Methods

### Data

#### Bathymetry and topography data

Bathymetric surveys in the MSC system have been done as indicated in Table 1. Spatial resolution varies between 0.25 m and 2 m. A Digital Terrain Model (DTM, 30 m resolution) was used to assess Baco-Malaylay catchments.

#### Core data

Several push cores and piston cores were collected in 2007 and 2017. Figure S1 shows cores location and seabed sediment characteristic grain size and sandiness from the 2017 survey.

#### Sub-bottom profiler

SBP data for shallow reflection seismic profiling were acquired in 2017.

East–West lines were acquired with a spacing of 80 m, whereas perpendicular North-South cross lines had 500 m spaced. The survey extends up to a minimum water depth of 50 m. A couple of lines following the path of the main canyon offshore the Malaylay River mouth were also acquired.

#### Rainfall and meteorological data

Rainfall data of Calapan weather station was obtained at different time scales (3 h, 6 h and 24 h) from the National Oceanic and Atmospheric Administration (NOAA) [www.ncdc.noaa.gov/cdo-web], and from the Philippine Atmospheric, Geophysical and Astronomical Services Administration (PAGASA). Data from other PAGASA stations in Oriental Mindoro (Villa Cerveza, Mahabang Parang, Poblacion-public market, Poblacion -San Teodoro, Malvar, Baras) have also been acquired and assessed. Recorded rainfall and meteorological parameters at Batangas weather station were also available, as well as several Climate Forecast System Reanalysis (CFSR) and Japanese Reanalysis Project (JRA) hindcast nodes in area of interest. Wind-induced error of rainfall gauge measurements during typhoons was bounded and accounted by the method described in^39^.

#### Typhoon hindcast data

Oceanweather Inc. (OWI) developed a tropical cyclone reanalysis hindcast for VIP which included significant typhoons within the 1951–2016 period. The available modeled typhoons are listed in Table S1. A continuous operational hindcast that covers a 1993–2012 period was also provided. Modeled parameters include significant wave height, period, direction, sea surface height, wind speed and direction, and depth-averaged current speed and direction from the ADCIRC + SWAN^40,41^ coupled hydrodynamic and wave numerical model, and OWI’s reanalysis of the wind fields associated with each storm^42^.

#### Typhoon tracks data

International Best Track Archive for Climate Stewardship (IBTrACS) data relevant for VIP was provided by NOAA [www.ncdc.noaa.gov/ibtracs]. IBTrACS data contain time evolution of typhoon path (latitude and longitude), associated wind speed and atmospheric pressure from several agencies monitoring tropical cyclones.

### Typhoon-induced waves and currents

The method to model the physical processes starting from typhoon induced conditions to turbidity current initiation and eventual development in the submarine canyons relies on coupling the typhoon hindcast with a sediment resuspension and transport model whose output is fed into the turbidity current model.

Sediment resuspension and transport due to the combined action of waves and currents at the shelf break is estimated following van Rijn *et al*.^43^. From cores and bed samples characteristic mud (20 μm) and sand (150 μm) sizes are defined. The method accounts for directionality of waves and currents and deviations from the Small Amplitude Wave Theory (SAWT) when computing orbital velocities^44^. Wave- and current-induced bed shear stresses are then used to derive the vertical distribution of suspended sediment based on Rouse profile for each sediment fraction (mud and sand). This process is shown in Fig. S2 for the period corresponding to typhoon Durian at a location on the shelf break near the head of the MSC shown in Fig. 1. This assessment clearly highlights the strong capacity of typhoon-induced waves and currents to resuspend sediment deposited around the canyon head. It also emphasizes that only during a limited time during the typhoon there will be significant volumes of sediment in suspension.

### Hyperpycnal assessment

A rainfall-runoff model was run to assess the main physical drivers of high-flow events in the Baco-Malaylay basin. The model accounts for soil moisture dynamics in a spatially distributed setting and incorporates information on the geomorphic complexity of river networks. The outputs of the hydrological model have then been coupled with a sediment transport model to assess whether these rivers could generate high sediment concentrations during major floods.

#### Hydrologic response of the Baco-Malaylay catchment

The model exploits information available on the landscape morphology, the land cover/land use and the underlying climatic forcing to represent, in a modular and spatially explicit framework, soil moisture dynamics in the root zone, groundwater storage variations, drainage from hillslopes and channel routing^45^.

The concept of “unit hydrograph” allows a proper spatial representation of hydrological processes especially for applications in poorly gauged areas, such as the Baco-Malaylay system, where no calibration is feasible and model parameters must be specified based on available information or climate and land properties^46^.

The retrieved datasets and main results are described in five categories for the basin topography and river network, the soil type and basin land-use/land-cover (LULC), climate and meteorological data, rainfall data and analysis of rainfall time-series, and application of the rainfall-runoff model to the Baco-Malaylay catchment (Supplementary Information: Hydrology).

The maximum water discharge predicted by the hydrological model in the assessed time interval is 210 m^3^/s for the Baco and 276 m^3^/s for the Malaylay. These conditions do not correspond to typhoon induced discharges. Discharges induced by Nock-Ten (Durian) rainfalls are estimated to be at most 157 m^3^/s (134 m^3^/s) for the Baco and 207 m^3^/s (177 m^3^/s) for the Malaylay.

#### Suspended sediment concentration at the river mouth

The BQART model, as described in Syvitsky and Milliman^29^, has been used to estimate yearly averaged sediment delivery of the Baco-Malaylay rivers to the ocean. In Supplementary Information: BQART model the specification of the various parameters used for the Baco-Malaylay system is discussed. Estimated total sediment yield for the Baco and Malaylay is 0.336 MT/year and 0.351 MT/year, respectively. This agrees with rivers that share the same lithology and relief temperature characteristics.

The BQART model quantifies yearly averaged sediment yield of a river, but it does not provide information on the inter-annual variability (extended droughts or major flooding) of the discharge-sediment load relationship, which is needed to predict the frequency and magnitude of hyperpycnal flows.

Direct monitoring of suspended sediment load was unavailable. Therefore, the hydrological modelling predicted instantaneous values of water discharge are coupled with a sediment transport model to provide estimates of the instantaneous values of suspended sediment concentration at the river mouth.

In the absence of a river bed survey, the transport capacity of the river has been estimated under the assumption of normal flow conditions. We applied a generalized form of the Engelund-Hansen^47^ sediment transport theory, as modified by Ma *et al*.^48^ to properly model the dynamics of fine-grained systems. This is described in Supplementary Information: Suspended sediment. It is estimated that at flood conditions Baco and Malaylay rivers might reach a suspended sediment concentration at the mouth no larger than 6 kg/m^3^.

#### Transformation of turbid river floods into hyperpycnal flows

River flow transformation into a turbidity current depends on the critical value of suspended sediment concentration^28^. A necessary condition for a river plume to plunge is then to have an excess density due to suspended sediments of at least 35–45 kg/m^3^, depending on the salinity and temperature of the coastal waters. Fresh river water has a typical density of ~1000 kg/m^3^, and coastal seawater a typical density of ~1025 kg/m^3^. Only a small number of rivers reach such a naturally high concentration of suspended matter.

If the critical concentration of 35–45 kg/m^3^ is assumed as a necessary condition to plunge, then hyperpycnal flows cannot be associated with flooding events of the Baco-Malaylay rivers, since for a wide range of assessed parameters the river suspended sediment concentration does not exceed 6 kg/m^3^.

It has been proposed that the critical concentration for initiation of hyperpycnal flows can be considerably smaller than 35–45 kg/m^3^, if processes of convective instability occur in the surface plume^49,50^. Sediment-driven convection has been shown capable of generating hyperpycnal flows at laboratory scale with a critical concentration of just 5 kg/m^3^ ^51^. However, direct observations of hyperpycnal flow formation from sediment-driven convection in a natural river plume have not been yet recorded. Besides, in the Baco-Malaylay system, the distance between the river mouth and the shelf-break (~250 m) is likely too short for the convective instability process to occur.

In summary, the modelling of the river flow response to the rainfall events in the Baco and Malaylay River catchment basins allows us to conclude that maximum river discharges associated with peak rain events do not reach high enough concentrations at the river mouths to generate hyperpycnal flows. Furthermore, among the group of largest floods during the period 2000–2017 there are events associated to monsoon rains, not just to typhoons. These results lead us to conclude that the turbidity currents were likely not triggered by hyperpycnal flows. It is possible, although unlikely, that during peak flood events the suspended sediment concentrations modelled at the river mouth reached an excess density of the order of 5 kg/m^3^, and may generate hyperpycnal flows via a re-concentration process associated with convective instability in the river plume as it enters the ocean. However, such hyperpycnal flows, if they occur, would have very low initial concentrations and velocities, and are unlikely to trigger strong turbidity currents down the MSC. Further details described in Supplementary Information: Hyperpycnal calculations.

### Turbidity current modelling

#### TCsolver model

Detailed computational fluid dynamics 3D numerical simulations were performed with TCsolver code. The mathematical formulation and extensive validation is described in several publications^52–58^. TCsolver employs a multiphase flow approach for modelling poorly sorted turbidity currents. It is governed by the mass and conservation equations for the incompressible bulk fluid, by the mass conservation equation of each particle size class and by the transport equation of mean turbulent kinetic energy *k* and its dissipation rate *ε*. Sediment entrainment is estimated employing the Garcia and Parker^25^ or the Smith and McLean^59^ methods. The solution of the governing equations requires the specification of physical boundary conditions at the computational domain edges. At the inlet, velocity, sediment concentration for each class, and the turbidity current thickness are specified as derived from metocean and sediment transport models (Methods: Typhoon-induced waves and currents).

Seabed sediment is modelled using two sizes, 20 μm mud and 150 μm sand. Initial seabed sediment composition is uniformly distributed in the computational domain and equal to 5% and 95% for each size respectively. These assumptions are based upon observations from the sediment cores. Critical conditions that give rise to sediment motion in the rock berm are assessed with Brownlie (1981) method^60^, corrected by local slope. Bedload transport rate is evaluated with the modified Meyer-Peter and Muller (1948) formula^61^ specifically calibrated for the rock berm sediment.

Further details on the modelling of turbidity currents associated with typhoons Durian, Melor and Nock-ten are found in Supplementary Information: Turbidity current modeling.

#### Sediment entrainment in the submarine canyons

The sediment entrainment relationship plays a crucial role in determining the spatial development of the turbidity current along the entire canyon system. We find the García and Parker equation to be a realistic choice^25^.

Based on available samples, the mud to sand ratio range (<5%) in the MSC upstream canyons does not have a significant effect on flow evolution. The turbulence closure in the numerical simulations has only a moderate effect on the results when the flow is not particularly strong, whereas it is more pronunced in stronger events.

### Sediment and seabed assessments

#### Turbidity current erosional patterns

**Figure 7:**
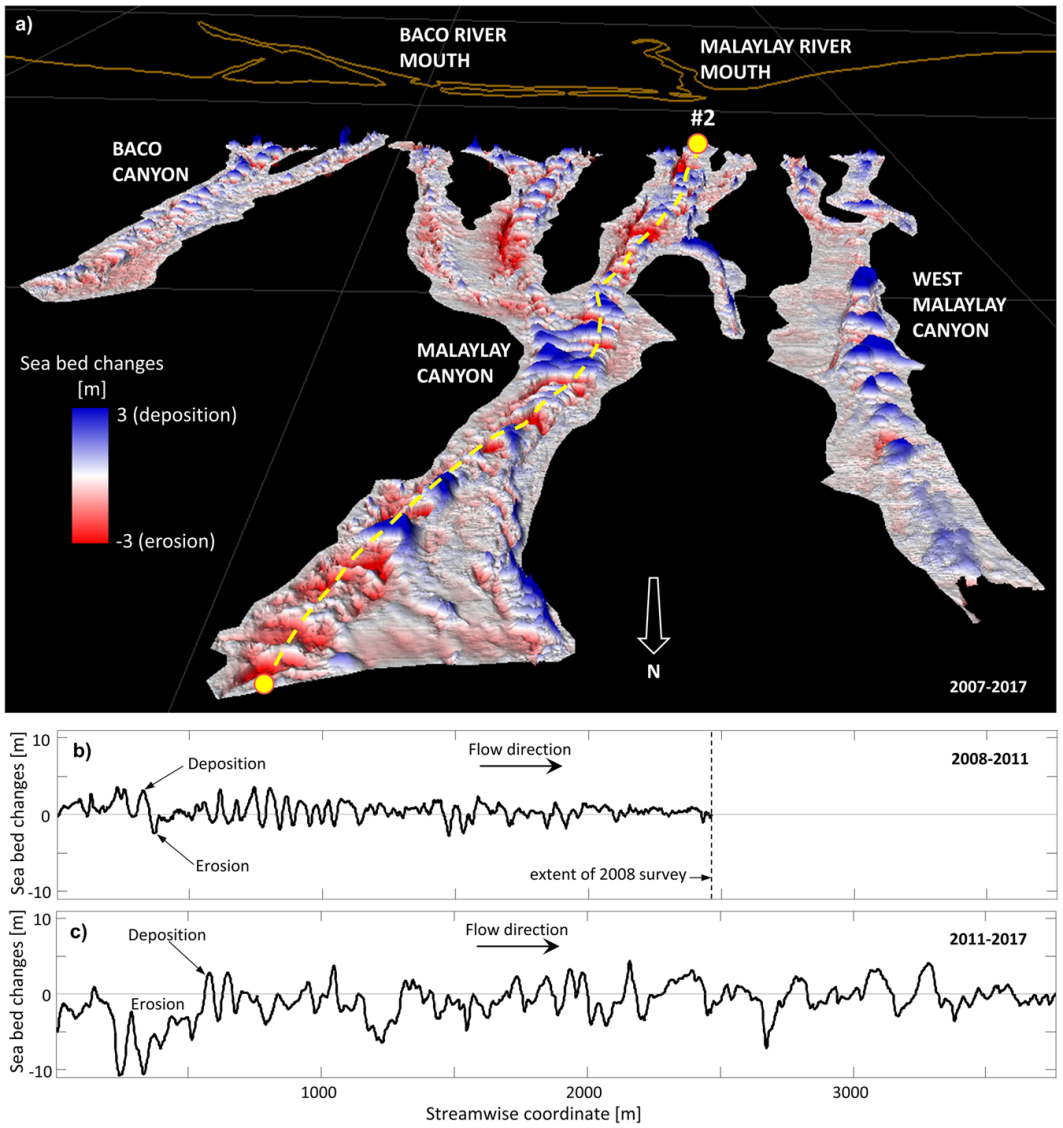
(**a**) In-channel erosion/deposition pattern as computed from the 2007–2017 difference map. Alternating bands of erosion (red) and deposition (blue) show that channels have been active during the 2007–2017 interval. Erosion/deposition difference maps along channel #2 for periods (**b**) 2008–2011 and (**c**) 2011–2017 indicate that sediment transport took place mostly after 2011 and the net balance was predominantly erosional.

The MSC is carved by numerous channels coalescent into three larger canyons. Channels that show clear bathymetric changes between different seabed surveys are interpreted as active. Trends of sediment accretion/erosion associated with turbidity currents can be assessed by available bathymetry difference maps. All major submarine canyons are active, but the MSC seems the most active of them all (Fig. 7a). During periods without large typhoon visits to VIP, such as 2007–2011, bedform activity is milder and vanishes downstream leaving a net depositional signature. Conversely the significant seabed changes between 2011 and 2017 spanning the entire length of the MSC are thought to be mostly related to the passing of Nock-ten. The balance is distinctly erosional, especially in the most upstream steep channels (Fig. 7c). Further analysis described in Supplementary Information: Sediment and sea bed.

#### Seabed activity inferred from STED

Following the first event in 2006 Self-Triggering Event Detectors (STED)^62^ were deployed at selected locations and times in the MSC and MC. None of these devices worked as expected, namely, by releasing a buoy to the surface that would transmit a signal to warn of a gravity flow event. Some were found out of position or damaged during periods when no large turbidity current was known to happen. The loss of STED provides further evidence of weaker flows in the canyons, although fishing activity cannot be ruled out.

## Supplementary information

Supplementary Information
