# Supplementary material for: How typhoons trigger turbidity currents in submarine canyons
Source: Sci Rep. 2019 Jun 25;9:9220. doi: 10.1038/s41598-019-45615-z (PMC6592950; doi:10.1038/s41598-019-45615-z)
Supplement: Supplementary file 1 — Supplementary Information [file 41598_2019_45615_MOESM1_ESM.pdf]

How typhoons trigger turbidity currents in submarine canyons

Octavio E. Sequeiros, Michele Bolla Pittaluga, Alessandro Frascati, Carlos Pirmez, Douglas G. Masson, Philip Weaver, Alexander R. Crosby, Gianluca Lazzaro, Gianluca Botter, Jeffrey G. Rimmer

Supplementary information

Core data

Figure S 1 shows cores locations and seabed sediment characteristic grain size and sandiness from the 2017 survey. Most data were taken from the MSC and the MC.

Typhoons

List of assessed typhoons

Table S 1. Most relevant typhoons capable of inducing significant oceanographic conditions in VIP, 1951-2016 period.

	IBTrACS Name / Storm ID	System Name
1	1951119N06145	IRIS
2	1952287N09148	TRIX
3	1952296N06152	WILMA
4	1957309N11161	KIT
5	1959356N05152	HARRIET
6	1960173N08137	OLIVE
7	1960273N10142	KIT
8	1966130N06139	IRMA
9	1967303N07150	EMMA
10	1970282N08147	JOAN
11	1970318N13157	PATSY
12	1971141N07156	DINAH
13	1977308N07156	KIM
14	1978288N10185	RITA
15	1981319N07163	IRMA
16	1981355N07149	LEE
17	1982227N09140	FAYE
18	1985284N05159	DOT
19	1987219N08134	BETTY
20	1987320N03171	NINA
21	1988005N03174	ROY
22	1988295N10138	RUBY
23	1988308N09140	SKIP
24	1989279N07151	DAN
25	1991163N11128	YUNYA
26	1993331N05172	LOLA
27	1994287N14156	TERESA
28	1995293N05177	ANGELA
29	1998285N12149	BABS
30	2006268N12129	XANGSANE
31	2006329N06150	DURIAN
32	2006340N08142	UTOR
33	2008169N08135	FENGSHEN
34	2013282N14132	NARI
35	2013306N07162	HAIYAN
36	2014190N08154	RAMMASUN
37	2014334N02156	HAGUPIT
38	2015344N07145	MELOR
39	2016_29_NWPAC	TOKAGE
40	2016_30_NWPAC	NOCK-TEN

Sediment resuspension and transport

The process to estimate sediment resuspension by waves and currents described in Methods: Typhoon-induced waves and currents is illustrated in Figure S 2. The capacity of typhoon induced waves and currents to resuspend sediment in the VIP trigger area drops abruptly with water depth. At 22 m water depth, near bed wave orbital velocities drop to half the magnitudes achieved at 10 m water depth. At 45 m water depth they drop to just 5%; below the critical orbital velocity to resuspend sand and just above threshold for mud resuspension. These estimations are made for peak typhoon conditions. From what follows that the periods during which resuspension is possible is further reduced in deeper waters. The effective zone where ignition of turbidity current is feasible by direct action of typhoon-induced waves and currents is thus confined to a narrow band.

Waves and currents direction

The shelf is narrow around Malaylay and Baco rivers mouth (~300 m wide), and refraction ensures wave direction to be mostly from the NW-NE range. Currents operate on a narrower band flowing toward either W-WSW or E-ENE (Figure S 3). Both tidal and residual components are shaped by the coast nearby. At the shelf break waves and currents are moving roughly perpendicular to each other.

Typhoon-induced currents and tidal currents

Typhoons whose paths lie close to VIP can induce currents larger than the regular tidal currents depending on water depth and on whether the typhoon occurs during neap or spring tides. At the shelf break typhoon induced currents can dwarf tidal currents by an order of magnitude, such as during Durian which arrived in VIP during a neap tide. More generally tidal currents can enhance or reduce residual (typhoon induced) currents depending on the timing of the storm, and on the relative direction of both currents.

Timing of river flood relative to waves

For Durian, Nock-ten and Melor and other relevant typhoons, peak wave conditions usually lag peak rainfall by about 3 hours (although in some cases peak waves occur before peak rainfall in VIP). In turn peak river discharge lags peak rainfall by about 12 hours.

Typhoons brought large rainfalls and river floods in the entire 2000-2016 timeseries (Figure S 6). However, rainfalls in the wet monsoon season can be more frequent or sustained and also result in substantial river floods. The wet summer monsoon brings heavy rains to most of the Philippines from May to October. The typhoon season occurs mostly between June and December. Summer monsoon and typhoon season therefore overlap, both phenomena contributing to larger river discharges during this period. As opposed to typhoon rains, monsoon rains are not usually associated with high winds and waves. Apart typhoons induced waves, wave climate is mild in VIP (Figure 3).

Typhoons with potential for t.c. triggering

Since 1951 there have been four events (Trix 1952, Betty 1987, Nina 1987, Durian 2006) well capable of exceeding the conditions for triggering turbidity currents. Nock-ten (2016), while having a smaller impact on VIP, must be also included in the list given the available evidence. The two confirmed cases are Durian, and Nock-ten. The characteristic of these five typhoons is that their eye passes through VIP (therefore just north of the trigger area) and that they are category 3 or larger on the Saffir Simpson scale (Figure S 4). A typical signature of waves and

currents for these typhoons is shown in Figure 3 corresponding to Durian. Waves and currents peak in tandem. Current direction during the most relevant period of the storm is towards the east. Typhoon induced (residual) currents overcome tidal currents by an order of magnitude.

In addition, there were another additional five events (Iris 1951, Dinah 1971, Roy 1988, Lola 1993, Hagupit 2014) which passed very close to the north of VIP and could have triggered turbidity current events had they been of a larger category at that location. For these events waves and currents peak mostly in tandem, but are not as strong as those from the first set of typhoons. Current direction is also towards the east. Typhoon induced currents are not much larger than tidal component.

Finally, there were another five events (Harriet 1959, Kit 1960, Irma 1966, Lee 1981, Melor 2015) that passed close but to the south of the trigger area, over northern Mindoro. A notorious case is Melor as currents experienced flow reversal at the same time waves were the highest (Figure 3). Current direction changes abruptly from eastward to westward. This change is driven by typhoon induced currents which dominate the total current regardless of the tidal component. The pipeline survey done afterward did not record any relevant seafloor change due to Melor in the VIP area. Harriet (1959) is also a special case because it travels toward the W-SW unlike the other four typhoons in this group which move towards the W-NW. This means that Harriet was closer to VIP during the waxing stage.

Flow reversal is mostly a function of the latitude at which typhoons pass near VIP, and characteristic zones can be identified based on the tracks and the metocean conditions at VIP. These are shown in Figure S 4 together with all critical typhoons within a 50-km radius of the trigger area.

As typhoon distance to the trigger area increases, the likelihood of trigger rapidly decreases. Nevertheless, it should not be discarded that smaller turbidity currents are triggered by typhoons which either cross the VIP area with low typhoon strength or pass further to the north or the south of the Malaylay River mouth with high typhoon strength.

Hydrology

Basin topography, contributing area and river network characterization

Figure 1 shows the topography of the area drained by the Malaylay and Baco rivers, whose catchment areas are 258 km^2 and 197 km^2, respectively. Mean altitude of the catchment is 766 m for Malaylay and 527 m. for Baco. Maximum relief is 1680 m for Malaylay and 2010 m for Baco.

Flow directions have been calculated following a maximum gradient algorithm. Subsequently the upstream area drained by each pixel in the DTM has been calculated based on the available flow paths. A minimum threshold has been imposed to the upstream cumulative area to individuate channelized sites and the shape of the river network. The overall number of sub-catchments for the Baco-Malaylay system is 46, which ensures a reliable description of the hydrologic response of the two rivers.

Basin soil type and LULC characterization

The characterization of the spatial distribution of soil type/order (i.e. lithology) and catchment LULC is a crucial ingredient for the rainfall-runoff model and can strongly affect the hydrologic response of the basin to precipitation.

The most general level of classification of soil is the Soil Order (www.usda.gov/). Soil orders are frequently defined by a single dominant characteristic affecting soils in that location (e.g.,

prevalent vegetation, type of material) or the climate variables (e.g., lack of precipitation, presence of permafrost).

Also, significant in several soil orders is the amount of physical and chemical weathering and/or the relative amount of pedogenic horizon development that has taken place. Following the guidelines provided by the Department of Agriculture of the Philippines (www.da.gov.ph/), Oriental Mindoro soil order is mainly represented by Ultisol, a particular soil with a fine texture caused by intense weathering occurring in humid climatic regions. Ultisols are good for agriculture and generally occur on old, stable, and highly weathered landscape positions such as those in sloping uplands, hills, and mountains where soils undergo fast weathering hastened by high temperature and high rainfall. These characteristics are distinctive of Oriental Mindoro island and Philippines archipelago, that has 27 % of its total land area occupied by Ultisols. As such, soil use in Malaylay and Baco catchments is largely expected to be dominated by cultivated area and moderate vegetation with a smaller fraction of deep forest.

To further strengthen the LULC classification of the area of interest, the results of an ad-hoc semi-automated remote sensing analysis on recently acquired multispectral Landsat-8 imagery have been also used. This satellite derived LULC analysis in combination with the data collected from publicly available sources were basin-upscaled and adjusted into four macro categories used as inputs for the rainfall-runoff model (see Figure S 5).

Climate and meteorological data

The meteorological data is from Calapan station (see Figure 1 for location) which provides for the period 2010-2017 daily averaged values of several meteorological variables useful for assessment of the potential evapotranspiration in the area. Specifically, mean, max and min temperatures slightly fluctuate around their long-term averages, which are 27°C, 29.6°C and 23°C respectively. Seasonality is indeed very low, but central months of the year (June to August) usually show higher temperatures (+10%) than other periods. Unfortunately, relative humidity measurements were not available at the Calapan station. Therefore, monthly averaged values provided by PAGASA (Philippine Atmospheric Geophysical and Astronomical Services Administration) have been used. Relative humidity is high throughout the whole year with very weak fluctuations around the long-term average which is 80-90%.

Rainfall data and analysis of rainfall time-series

Rainfall patterns are characterized by high spatial and temporal variability. The accurate description of these patterns is important in modelling the hydrologic response of a catchment, because the hypothesis of spatially homogeneous rainfall is only valid when the area of the catchment is smaller or comparable with the macroscale of rainfall processes.

The overall size of the Malaylay-Baco catchment (~500 km^2) would have required the construction of spatially distributed rainfall fields to efficiently incorporate not only the temporal but also the spatial variability of rainfall within the catchment. Unfortunately, assessing the spatial variability of rain in the catchment was unfeasible because of the limited quality of the available rainfall data at the various rain stations. All possible sources of rainfall data within and nearby the area of interest have been surveyed.

Rainfall data made available to the public by PAGASA (6 stations) have not been considered because the data are too limited (time-series starts in 2013) and of poor quality. In contrast, Calapan station rainfall data have been measured since the 1950’s and are available at different time scales: 3h, 6h and daily. They are considered as three different time-series with distinctive sample size and completeness, the key issue being the presence of ’no data’ in the timeseries, which affects more than 50% of the original 3h dataset. This percentage reduces when analysing 6h or 24h datasets, in particular after the year 2000, when rainfall depths can be considered

complete and reliable. Whilst floods can be efficiently reproduced in terms of overall flow volumes even with 24h rainfall data, the peak flow would however be missed without any information on how daily rainfall volumes are distributed in time at higher frequency. For this reason, available 6h and 24h rainfall datasets have been downscaled to provide completeness to the 3h dataset assuming spatially uniformity.

Rainfall analysis includes the fitting of probability density function on the annual maximum of rainfall (Generalized Extreme Value distributions, GEV) over the three duration datasets, and the identification of the intensity-frequency-duration (IFD) curves. The region is affected by significant rainfall events, with more than 600 mm/day (and 200 mm/3h) that are expected (on average) once a century. The IFD curves were then used to estimate the return periods of selected rainfall events corresponding to a set of relevant typhoons which occurred from 1977 to 2016. Data show that most typhoons are characterized by relatively low (i.e., <10 years) return periods for all the durations explored. The likely reason is twofold: on one hand, significant rain events are observed in the area also during events that are not classified as typhoons; on the other hand, the wind speed (that might instantaneously exceed 250 km/h) represents a source of error on rain measurements during typhoons.

Application of the rainfall-runoff model to the Baco-Malaylay catchment

The application of the hydrological model to the Baco-Malaylay rivers to estimate hydrographs at the outlet of the study catchments required the specification of a certain number of physical parameters that can be grouped in three main classes: soil and vegetation, flow, and hydrodynamic parameters.

The values assumed by soil and vegetation parameters were derived from the literature, considering the underlying soil type and vegetation cover in the study area. Flow parameters were instead derived from hydrologic data in nearby catchments, where synchronous daily rainfall/runoff data were available. Hydrodynamic parameters known to bear a negligible effect on the shape of the hydrograph in catchments whose size does not exceed some thousands of km^2, were estimated based on judgement and literature review. A comprehensive list of the inputs parameters is described by Rodriguez-Iturbe and Rinaldo[SI-2].

The timeseries of modelled discharge for the Baco and Malaylay catchments during the entire simulation period is shown in Figure S 6. The rainfall signal was cautiously increased (doubled) in correspondence of both Durian and Nock-Ten events to compensate for potential underestimation of rain measurements because of high wind speed. According to model simulations, flow regime in the Baco-Malaylay River system is highly dynamical and erratic, with sudden and pronounced increments of streamflow in response to rain events, and prolonged dry periods. The hydrologic response of the study catchments comprises multiple interacting timescales: i) a rapid response with a typical duration of 5 to 10 hours, related to fast surface runoff, preferential flows and drainage of highly saturated soil; ii) an intermediate response with a typical duration of 48 hours, mainly related to the drainage of the topsoil layer and the root zone; and iii) a slow response with timescales of about 10 days, which relate to the groundwater contribution to discharge, and can be explained by the drainage of the aquifer during relatively dry periods.

The flooding potential of the area turns out to be quite high and not restricted to typhoon events. Large floods are indeed induced by rainfall events bringing significant water volumes when the antecedent moisture of the catchment is relatively high. Conversely, the study catchments seem to be less sensitive to shorter (but more intense) events.

The effect and the importance of both the soil moisture of the catchment prior to the event and the duration of the rain event is further evidenced by the response of the system to both Durian and Nock-ten typhoons. Durian was indeed preceded by a very dry period, which caused the soil moisture of the catchment to approach the wilting point and the resulting hydrological response

to be quite smoothed. Compared to Durian, Nock-ten occurred after a month characterized by a moderate amount of rainfall. In this case, however, the hydrological response is damped by the duration of the rain event which was not sufficient to saturate the residual storage capacity of the catchment soil, thereby preventing the whole catchment area to be simultaneously active in contributing streamflows to the outlet. Return periods of river discharges associated with recent typhoons have also been analysed and shown to be quite limited (~5 years) in terms of peak flows.

BQART model

The *B* factor addresses glacial erosion, lithology, lake and reservoir trapping, and human-induced erosion. The absence of glacier cover, together with the negligible presence of sediment traps (e.g. lakes and reservoirs) or other significant anthropogenic disturbances (e.g. mining activities) within the area of interest, set the specification of the *B* factor solely dependent on the average Baco-Malaylay lithology. Following the classification study of Syvitsky and Milliman[28], the Philippines region can be considered as predominantly composed by mixed-carbonates, volcanic rocks. Because of all these considerations, $B = 1$ was thought to be a reasonable choice for Baco Malaylay catchments.

Based on modelling results (Figure S 6), the Baco River has a long-term yearly averaged water discharge of 9.84 m^3/s, whereas the Malaylay River reaches a slightly higher value of 13.09 m^3/s. Figure S 7 shows basin-averaged discharge with drainage area, with its fitting power-law relationship and superimposed runoff (Q/A) isolines. Both Baco and Malaylay plot nicely in the region of small-sized basins characterized by high runoff.

Estimated total sediment yield for the Baco and Malaylay is 0.336 MT/year and 0.351 MT/year, respectively. Figure S 7 shows sediment yield trends as a function of drainage area as derived from the global database[28]. Red dots represent rivers that share the same lithology factor and have a similar product *RT* (relief, temperature) to the Baco-Malaylay system.

Suspended sediment

Ma et al.[56] sediment transport method is based on flow conductance, Shields number, and two coefficients function of the sediment grain size. Besides the instantaneous water discharge (output of the hydrological model), the following quantities need to be specified: channel width and bed slope, characteristic sediment grain-size, and flow conductance.

The channel width is estimated from satellite images focusing on the portion of the rivers, right upstream the river mouth, where limited streamwise variation of the channel width is present. In this region, where normal flow conditions are assumed to apply, the Baco River characteristic width is 45 m, whereas that of the Malaylay is 50 m.

The longitudinal bed slope is computed, in the same region where the channel width was estimated, extracting the streamwise variation of bed elevation from the available DTM. The resulting longitudinal gradient for both Baco and Malaylay is approximatively 0.0057°.

Core samples obtained from the offshore areas show that sediment in the canyons is sand, typically about 100-200 μm. Mud is, however, present on top of the sand layers, and on the flanks of the canyons, and possibly also distally beyond the study area. The quantification of the fraction of mud in the sediment transported by the river is however an uncertain task, and, in the absence of direct measurements, scenarios of mud dominated (finer than 60 μm) suspension are also considered in the analysis. A range of sediment size from 20 to 200 μm has been explored.

Based on the above parameters the conductance is estimated considering the scenario of peak flow discharge. Under these conditions, the river bed configuration is estimated to be covered by dunes. The formulation proposed by van Rijn (1984)[SI-3] is then employed to evaluate the total (i.e. skin and form drag) flow conductance, predicting a range of 15-20 for both Baco and Malaylay rivers.

Assuming sediment discharge does not vary from the region where normal flow approximation holds till the river mouth, the instantaneous value of the depth-flux averaged suspended sediment concentration at the shoreline is then computed as the ratio between sediment load provided by the sediment transport model and water discharge provided by the hydrological model. Figure S 8 shows the behavior of the predicted suspended sediment mass concentration C_{shore}^{m} as a function of outlet flow discharge for given sediment grain-size and flow conductance. The coarser the material to be transported is, the lower the suspended sediment concentration at the outlet is. In contrast, an increase in flow conductance C_z directly translates into an enhanced total suspended load.

Hyperpycnal calculations

Hyperpycnal flows occur when river density is larger than the ocean's. Under these circumstances, a turbid plume can plunge to form turbidity currents. The critical mass concentration for plunging is

$$C_{cr}^{m} = \rho_s C_{cr} = \frac{\rho_s}{R_g}\left(\frac{\rho_a}{\rho_w} - 1\right)$$

where ρ_s, ρ_w, ρ_a are the density of the sediment, the river clear water, and of the sea water, respectively. R_g equals to $(\rho_s - \rho_w)/\rho_w$. C is the concentration (in volume) of suspended sediment in the river. Figure S 9 shows the modelled time-series of the suspended sediment concentration at the Malaylay River outlet as predicted by coupling the results of the rainfall-runoff model and the sediment transport model. In the computation, flow conductance C_z has been set equal to 17.5. Critical triggering conditions cannot be reached by either Baco or Malaylay rivers even when a totally mud dominated scenario (i.e. d_s =20μm) is considered.
A further estimate of the yearly averaged sediment yield of the Baco-Malaylay system, alternative to the prediction provided by the empirical BQART model, can be computed by integrating and yearly averaging the modelled time-series of the sediment discharge at the river outlet. Figure S 10 shows the behaviour of the sediment yield of Malaylay River as a function of sediment grain size and for different values of flow conductance. For a suspension having a characteristic grain size ranging between 70-130 μm, the averaged total sediment yield for the Baco and Malaylay is 0.70MT/year and 0.95MT/year, respectively. These values are a little higher but indeed compatible with BQART empirical predictions and sit nicely within the global database when plotted against basin drainage area (Figure S 7).

Turbidity current modeling

The flow originates in the southern boundary of the computational domain at the shelf break in approximately 10 m water depth, consistent with the location of the hindcast node of waves and currents. Table S 2 reports the main input conditions for turbidity current modelling of Durian, Melor and Nock-ten. All TCsolver simulations were run in three-dimensions.

Table S 2. Summary of specific model inputs for selected turbidity current simulations.

	Durian	Melor	Nock-ten
Number of grain sizes	2	2	2
Sediment grain size	20 μm, 150 μm	20 μm, 150 μm	20 μm, 150 μm
Initial seabed composition	5%, 95%	5%, 95%	5%, 95%
Max. inlet volumetric concentration	5.60 10^{-3}, 2.5 10^{-4}	1.74 10^{-3}, 5.11 10^{-5}	1.64 10^{-3}, 5.32 10^{-5}
Max. inlet velocity along x, y, z	1.27, 0.64, 0 m/s	-0.10, -0.05, 0 m/s	1, 0.48, 0 m/s
Inlet thickness	2.8 m	2.8 m	2.8 m
Seabed form roughness *	0.075 m	0.075 m	0.075 m
Seabed skin roughness	200 μm	200 μm	200 μm
Turbulence model	Mellor & Yamada	Mellor & Yamada	Mellor & Yamada
Sediment entrainment relation	Garcia & Parker	Garcia & Parker	Garcia & Parker

* This form drag roughness accounts for sub-grid scales, as the larger bedforms wave-lengths are much longer than the grid size.

Modelling of Durian turbidity current (2006)

According to the model during the turbidity current triggered by typhoon Durian all small upstream canyons of the MSC were activated (Figure 4). Ignition of large turbidity currents is enabled when the orientation of the upstream canyon axes is matched by the longshore current, which in the case of Durian is toward the E-NE (~63°) and provides a rate of inflow of momentum through the canyon's heads.

The current is confined within channel sidewalls in the most upstream stretches of the MSC system, then it overspills on the left side before crossing the pipeline for the first time at the "bend", where the pipeline was shifted laterally by 110 m (Figure 1). At the "bend" the predicted depth-averaged velocity is above 5.5 m/s. In the MC the current is redirected toward the northwest, almost parallel to the pipeline.

At the "narrowing" a local increase in longitudinal bed slope together with the funnelling of the canyon further accelerates the flow, reaching a depth averaged value of about 6 m/s. The current is here diverted by the right sidewall towards the pipeline, crossing it at an angle of approximately 30° where the pipeline was shifted laterally to the left by 42 m. On the left side, some overspill is observed. Further downstream, the channel widens and the current decreases in velocity.

The time required for the current to reach the "narrowing" starting from shallow water is approximately 25 min. Total length travelled is about 7.5 km.

Modelling of Nock-ten turbidity current (2016)

Despite inflow conditions being forced along the entire shelf break the current is triggered with a significant flow velocity only along channel #3 located between the Malaylay and Baco River mouths. The reason why not all upstream canyons in the MSC system are activated seems to be related to the orientation of each canyon axis with respect to the longshore current that, in this case, flows toward the E-NE (~64°) rather than differential upstream forcing or different channel gradients. The head of channel #3, which hosts the turbidity current, is oriented in such a way that the longshore current provides a momentum flux at its upstream end, whereas the other channels only receive a portion of it. A higher resolution hindcast model might provide more variation in current directionality across the shelf, which might result in additional upstream channels being activated.

Once confined in the main branch of the MSC the current follows a similar path than Durian's, albeit slower and thinner. There is also less overspill onto the levees. At the "narrowing" the current crosses the pipeline at an angle of approximately 10°-20°. This is the location were the berm was damaged after typhoon Nock-ten. The time required for the current to reach the "narrowing" starting from shallow water is approximately 35 min, longer than Durian's.

Figure 4 shows the erosion/deposition map of the rock berm at the "narrowing" based on bathymetry surveys before and after Nock-ten. Also shown is the modelled Shields stresses exerted on the seabed by the turbidity current. Stress is relative to the critical threshold of transport of rock berm material (eclogite, 3200 kg/m^3, 13 cm mean diameter).

At the "bend" erosion of the upstream side of the rock berm is likely associated with the reduction of the critical Shields number due to the large lateral slope of the edges of the rock berm. Conversely, erosion on the downstream side of the berm is due to an increase in shear stress on the adjacent area due to the presence of a bedform on the seabed.

Modelling of Melor turbidity current (2015)

TCsolver simulations run with typhoon Melor (2015) peak inflow conditions showed that they were not sufficiently intense to trigger any significant turbidity current down the MSC. Model results are confirmed by bathymetry difference maps between 2016 and 2015 surveys showing that only very modest seabed activity occurred around the "narrowing" and that there are no evidences of erosion or deposition areas along the rock berm.

Determination turbidity current duration

Once the ignition boundary is defined (3.1) the time period over which conditions are suitable for turbidity current ignition define a first order estimation of turbidity current duration. With the caveat that the actual duration might also be limited by sediment availability in the canyon the predicted durations are in Table S 3.

Table S 3: Typhoons that likely triggered large turbidity currents in Malaylay Submarine Canyon.

Typhoon (year)	Duration [hr]
Trix (1952)	2.75
Harriet (1959)	1.75
Betty (1987)	3.00
Nina (1987)	1.75
Durian (2006)	3.25
Nock-ten (2016)	0.75

Sediment and sea bed

Sediment budget at the MSC head

The MSC head is located just to the north of the Malaylay and Baco river mouths. Sediment supply from the rivers is therefore the likely most relevant sediment source. The shelf feeding the MSC system has an area of roughly 3 km by 0.25 km and is considered as a control region for the sediment budget analysis.

Given the usually mild wave and moderate tidal conditions, sediment load from the rivers is prone to deposit on the nearshore. As described in Methods: Hyperpycnal assessment the minimum annual sediment supply from the Malaylay and Baco rivers is estimated to be 1.65 MT/y. If all sediment coming from the rivers is assumed to be trapped in the control region, the resulting yearly vertical accumulation of the sediment would be ~1.38m (assuming porosity 0.4). This is a maximum thickness, since sediment from the rivers is likely deposited in a broader area, being spread by longshore currents.

Assuming the longshore sediment entering the area approximately balances the longshore sediment leaving it, we estimate the total sediment supplied to the canyon head by a 3.25 hours turbidity current triggered by typhoon Durian to be 0.52MT, about 31% of the yearly sediment discharge of the rivers. Thus, the sediment supply from the Baco and Malaylay rivers is of the same order of magnitude as the total sediment volume required to sustain a large turbidity current event per year.

Turbidity current erosional patterns

Figure 7 shows the 2017-2007 bathymetry difference map. Signatures of channels activity (i.e. alternating bands of erosion and deposition), which took place between 2007 and 2017 are evident. Channel #2 is arguably the most active. Signatures of channel activity are present during both 2007-2008 and 2008-2011 time intervals. However, the amplitude of these alternating bands

of erosion and deposition are small if compared with 2011-2017 activity and vanishes in the more distal part of the slope. The activity of the seabed seems maintained by weaker flows, whether of tidal origin or smaller turbidity currents, that do not runout completely down the slope. Erosion and deposition mainly occurred after 2011. This activity in the upstream branches of the MSC could have taken place at any time during the 2011-2017 interval, but arguably most of the changes occurred after the passage of typhoon Nock-ten, as observations on the more frequently surveyed MC suggest.

References for supplementary information

SI-1. Komar, P.D. & Miller, M.C. Sediment threshold under oscillatory waves. *Proc. 14th Coastal Engineering Conference.* Copenhagen, **2**, 756-775 (1974).
SI-2. Rodriguez-Iturbe, I. & Rinaldo, A. *Fractal river basins.* (Cambridge University Press, 1997).
SI-3. van Rijn, L.C. Sediment Transport, Part II: Suspended Load Transport. *Journal of Hydraulic Engineering*, ASCE, **110**, 11 (1984).

Figures for supplementary information

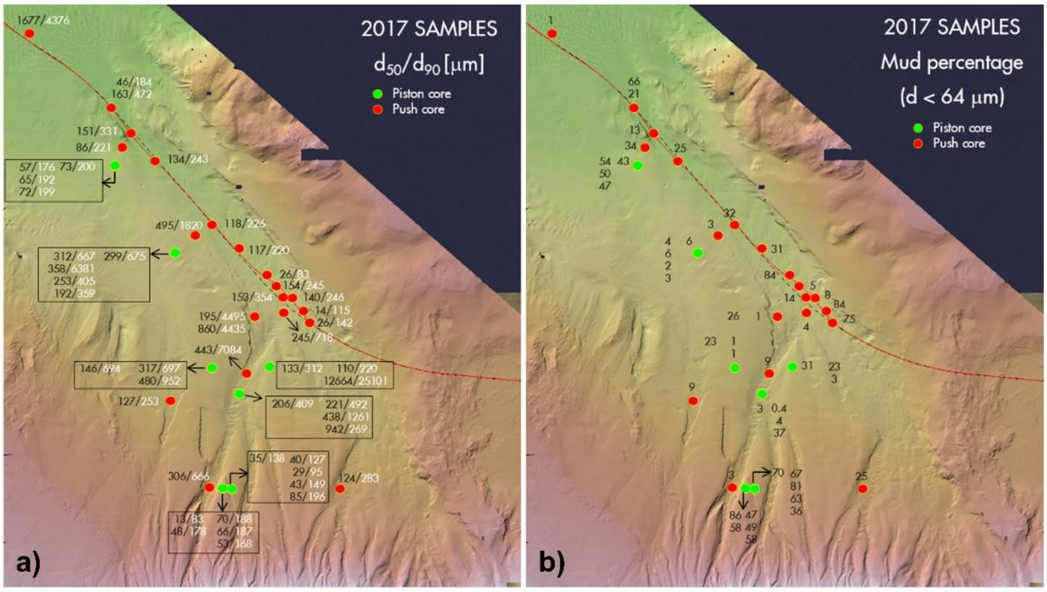

Figure S 1: Sediment samples collected during the 2017 campaign with push (red dots) and piston (green dots) cores. a) Values of d_{50} (black font) and d_{90} (white font) in microns as obtained from grain size analyses. When multiple values are present in the same location, they refer to grain size analyses performed at different elevations below the seabed. b) Percentage of mud content in the sediment sample. Sediment is classified as mud when diameter is below 64 μm.

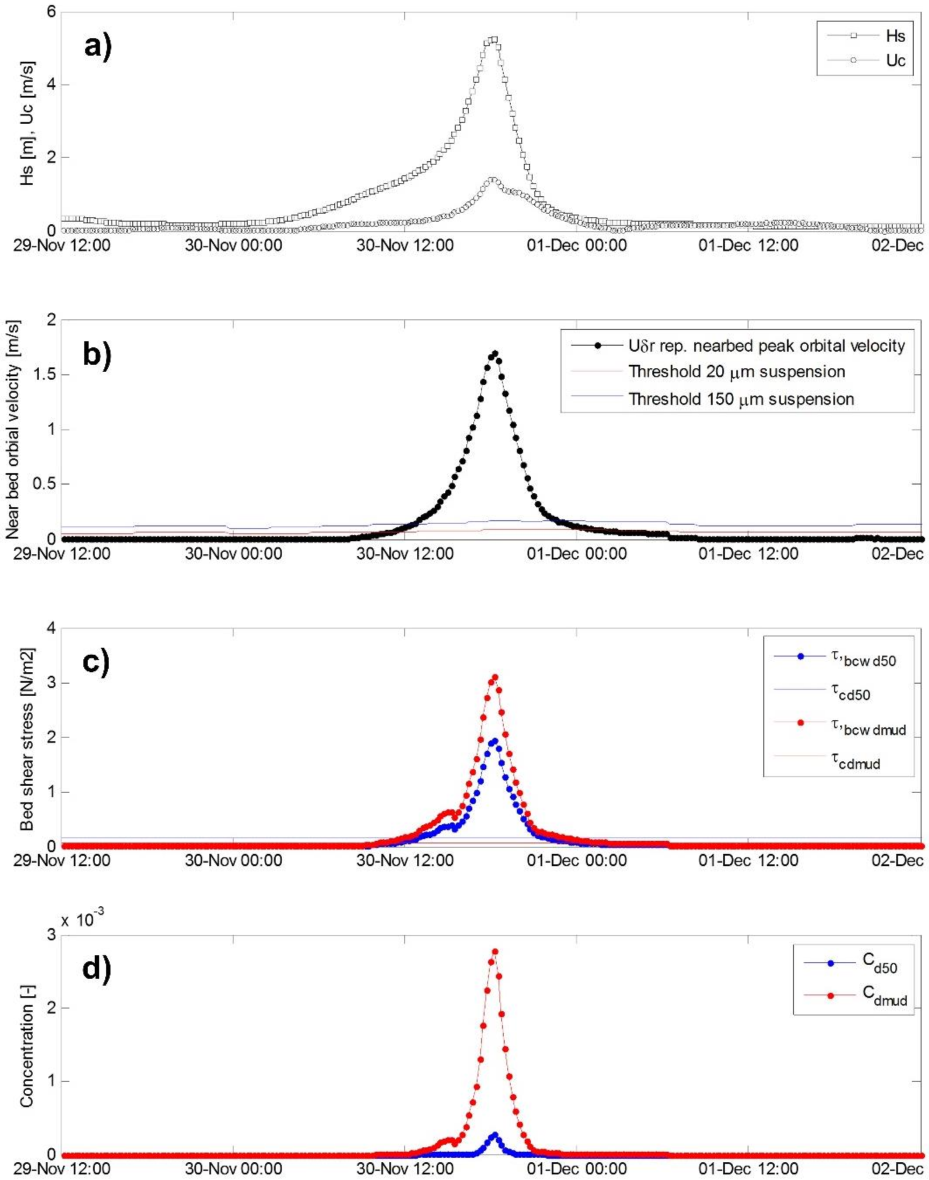

Figure S 2: Translating wave and current conditions from the typhoon hindcast into suspended sediment concentration. Time interval corresponds to typhoon Durian. Hindcast node is at the shelf break in water depths of about 10 m (Figure 1). a) Waves and currents. b) Near bed peak orbital velocity and threshold orbital velocity of suspension for mud and sand (Komar and Miller[SI-1]). c) Effective combined bed shear stress from currents and waves for mud and sand, and critical shear stress for mud and sand. d) Suspended sediment concentration of mud and sand.

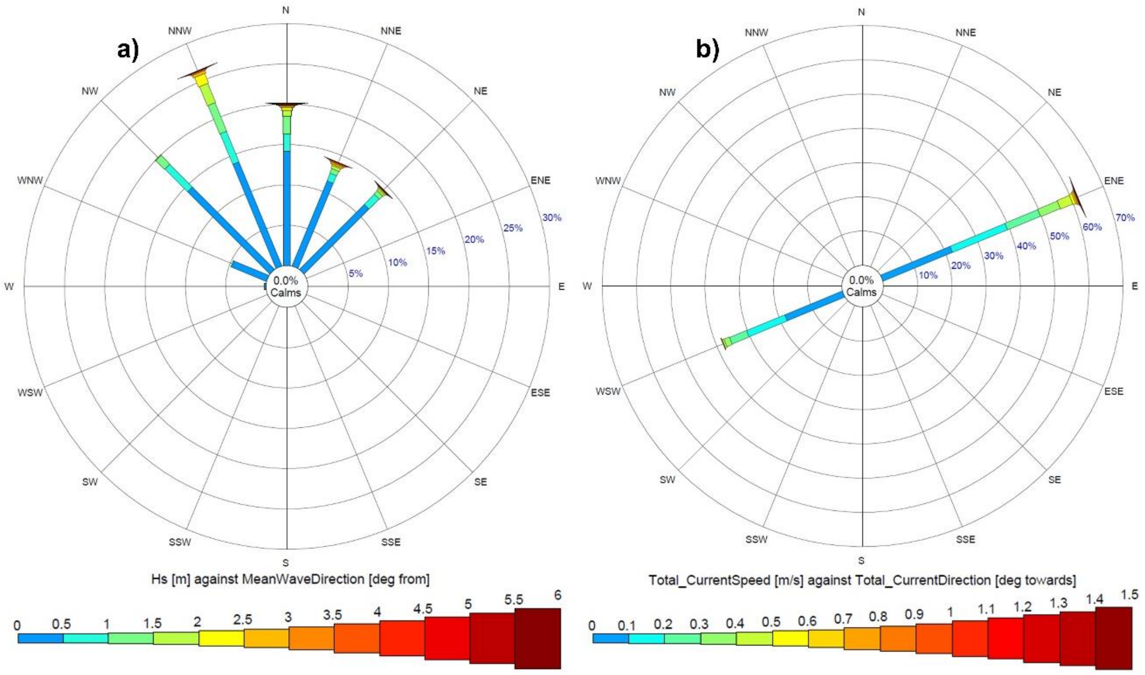

Figure S 3: a) Wave and b) current roseplots for all typhoons in the hindcast database (Table S 1). See precise node location at shelf break in Figure 1.

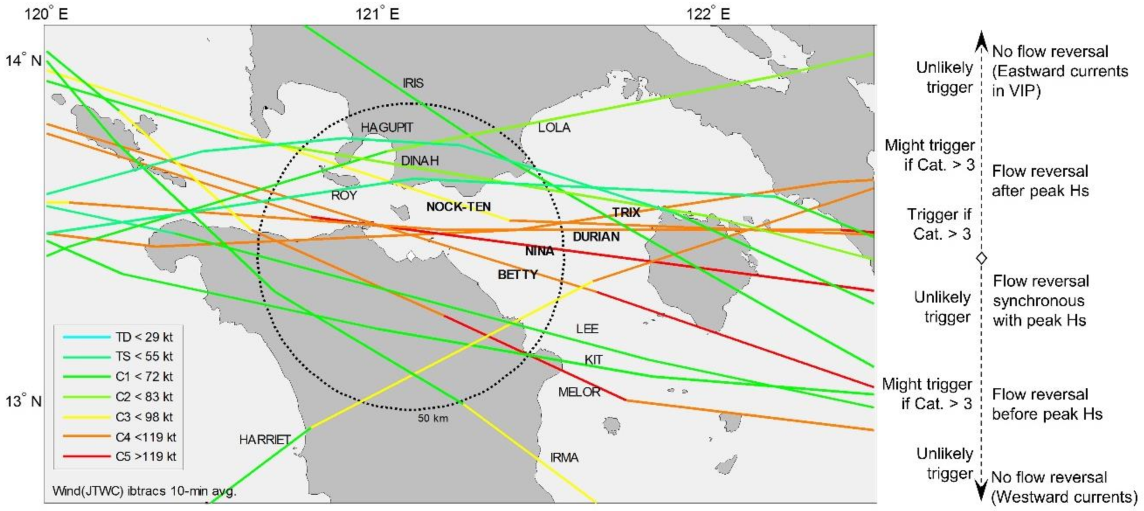

Figure S 4: Track of relevant typhoons near VIP trigger area, indicated with a white diamond. Legend represent typhoon category based on Saffir-Simpson scale. Shown on the right are the estimated conditions for triggering large turbidity currents in MSC as a function of distance to the trigger area; and are approximate zones where typhoon tracks result in different current conditions in VIP.

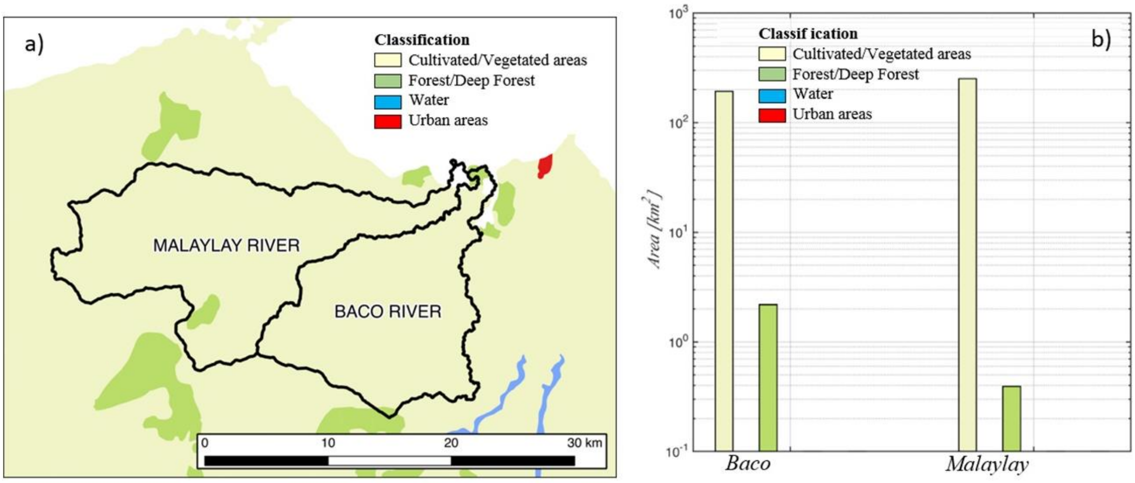

Figure S 5: a) Upscaled soil use classification of north Mindoro. b) Areal occupancy of each soil use class in the Baco and Malaylay rivers catchments as applied for rainfall-runoff modelling.

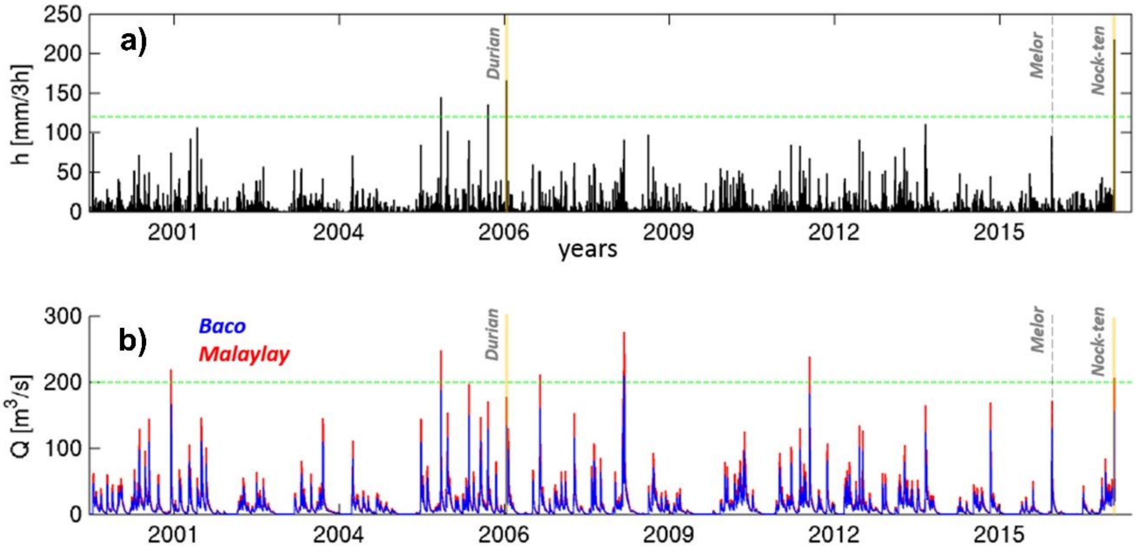

Figure S 6: a) Rainfall time-series reconstructed from Calapan 3h measured data. Note that, in correspondence of Durian and Nock-ten events (light orange vertical lines), rainfall signal has been doubled to compensate for potential underestimation of rain measurements due to high wind speed. As a reference, also Melor typhoon (grey dashed vertical line) passage above VIP is reported. b) Modelled water discharge at catchment outlet during the entire simulation period (2000-2017).

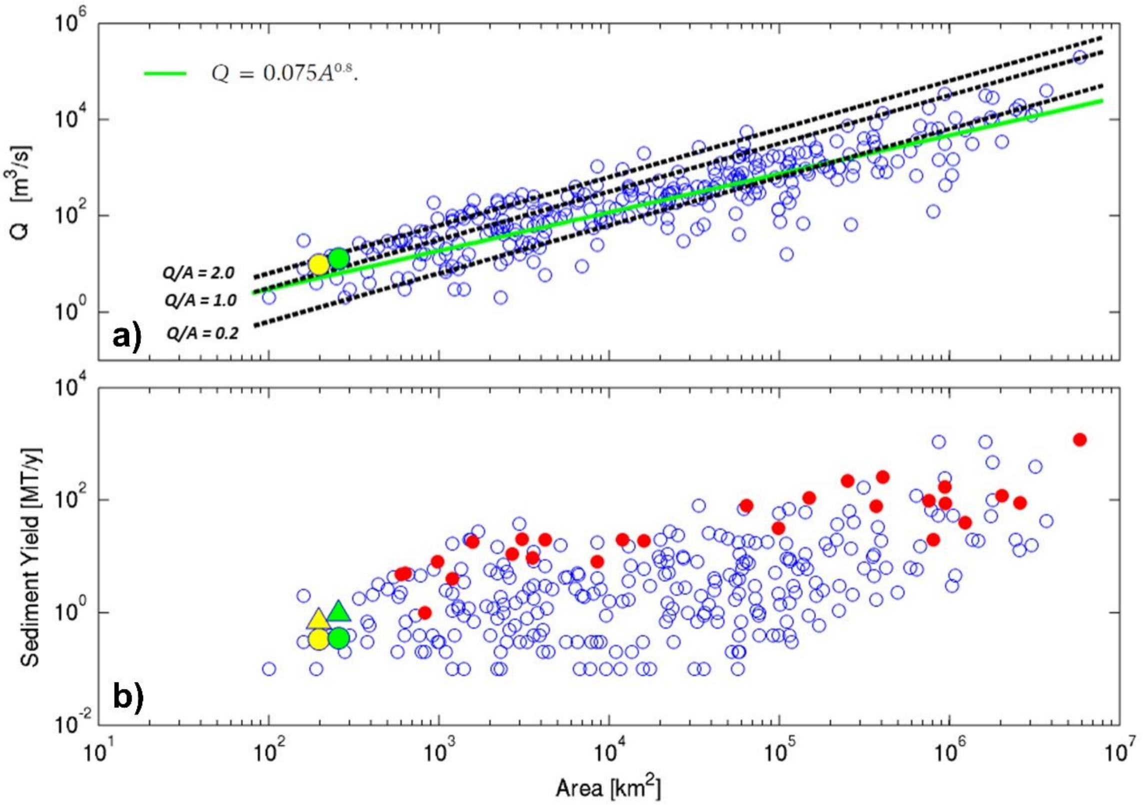

Figure S 7: a) Basin-averaged discharge with drainage area, with fitting power-law relationship and superimposed runoff *Q/A* (m/y) isolines. Baco (yellow dot) and Malaylay (green dot) catchments belong to the small-sized basin domain with high-runoff. b) Basin-averaged sediment yield with drainage area. Red dots represent rivers that share the same lithology factor and have a similar product *RT* (relief, temperature) of the Baco (yellow dot) and Malaylay (green dot) systems. Triangles represent Baco (yellow) and Malaylay (green) sediment yield estimates computed from the modelled time-series of sediment discharge (Methods: Hyperpycnal assessment).

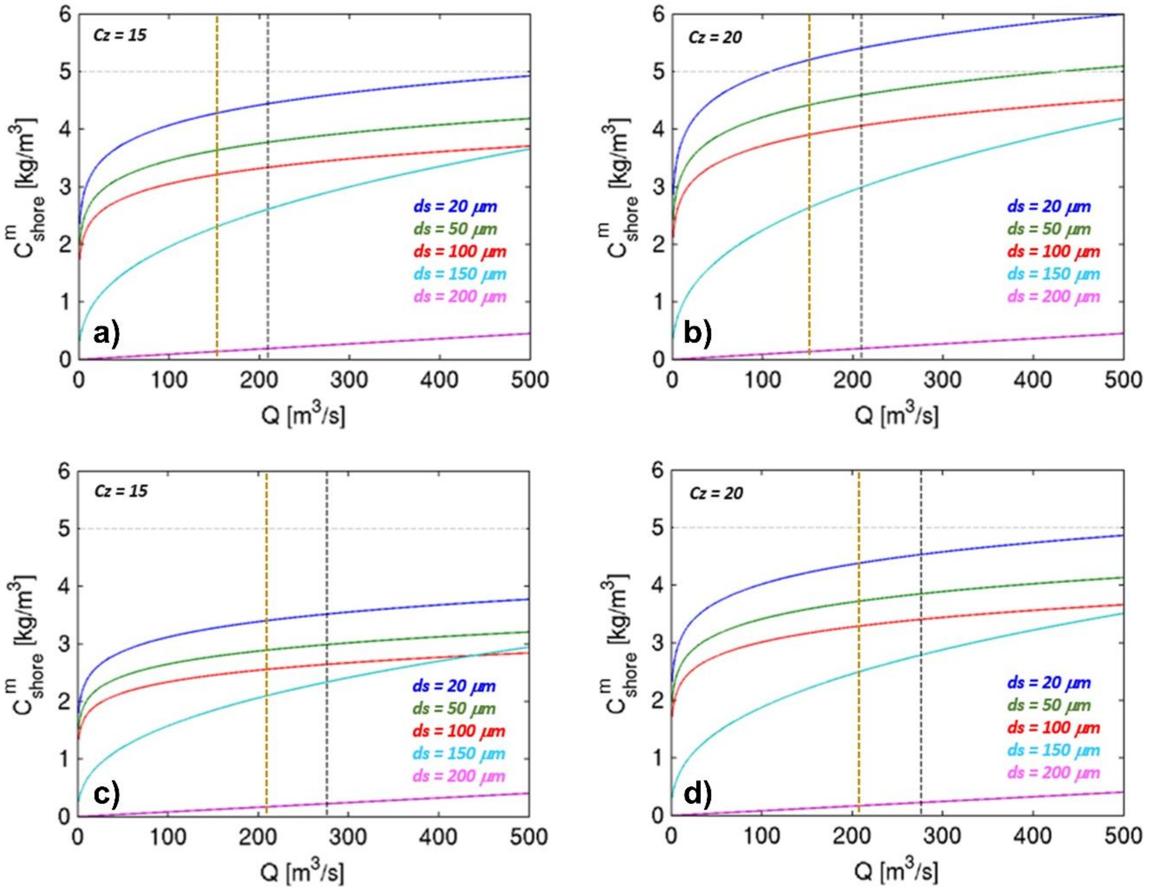

Figure S 8: Predicted sediment concentration at the river mouth C^m_{shore} as a function of water discharge Q for given mean grain-size and flow conductance C_z. Upper panels (a, b) are predictions for the Baco River, and lower panels (c, d) for the Malaylay River. In each panel: the black vertical dotted line indicates the maximum water discharge predicted by the hydrological model in the modelled time interval (~210m^3/s for the Baco and ~276m^3/s for the Malaylay); the brown vertical dotted line represents maximum modelled discharge during Nock-Ten when doubled rainfall signal has been considered (~157m^3/s for the Baco and ~207m^3/s for the Malaylay); the grey horizontal dotted line represents the threshold concentration for formation of hyperpycnal flows in the case of re-concentration processes.

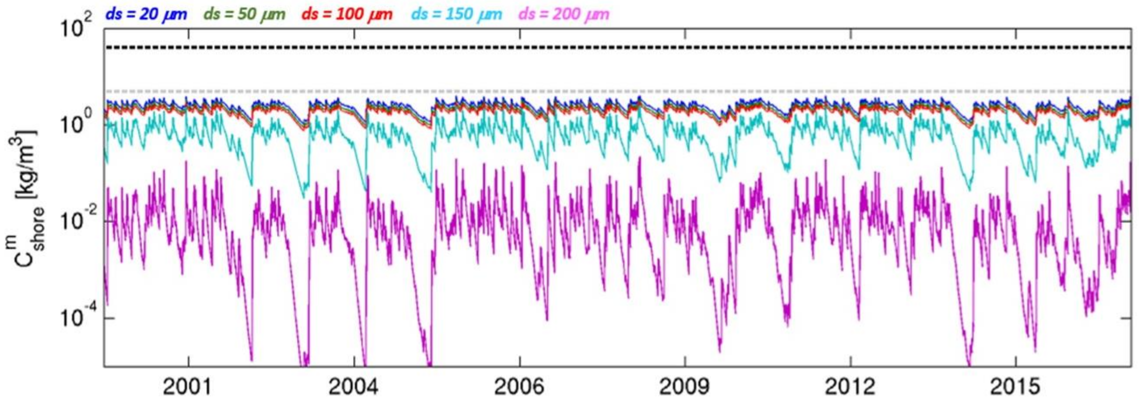

Figure S 9: Time-series of the suspended sediment concentration at the Malaylay River outlet as predicted by coupling the results of the rainfall-runoff model and the sediment transport model. In the computation flow conductance C_z has been set equal to 17.5. Predicted sediment concentrations are always below the critical value for plunging (black dotted line) also when re-concentration processes are accounted for (grey dotted line). Baco River time-series (not shown) are similar.

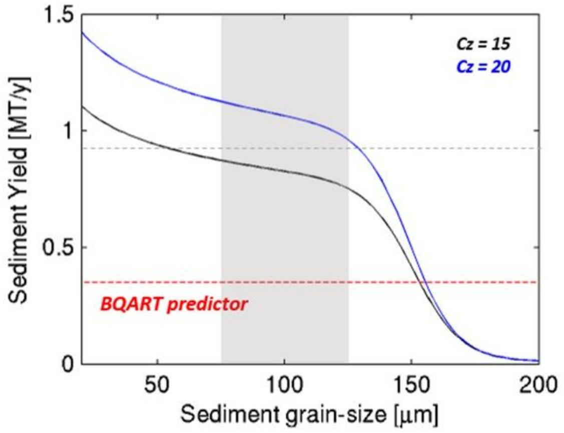

Figure S 10: Total sediment yield for the Malaylay River as a function of sediment grain size and for different flow conductance, computed by coupling the output of the hydrological model and the sediment transport model. Grey dotted line represents the average sediment yield for a suspension having a characteristic grain size range of 70-130 μm. Red dotted line represents the sediment yield as estimated from the BQART model. Baco River (not shown) has similar trend.
